# Proteomics of Heat-Stress and Ethylene-Mediated Thermotolerance Mechanisms in Tomato Pollen Grains

**DOI:** 10.3389/fpls.2018.01558

**Published:** 2018-11-12

**Authors:** Sridharan Jegadeesan, Palak Chaturvedi, Arindam Ghatak, Etan Pressman, Shimon Meir, Adi Faigenboim, Nicholas Rutley, Avital Beery, Arye Harel, Wolfram Weckwerth, Nurit Firon

**Affiliations:** ^1^Institute of Plant Sciences, Agricultural Research Organization, Volcani Center, Rishon LeZion, Israel; ^2^The Robert H. Smith Faculty of Agriculture, Food and Environment, The Robert H. Smith Institute of Plant Sciences and Genetics in Agriculture of The Hebrew University of Jerusalem, Rehovot, Israel; ^3^Department of Ecogenomics and Systems Biology, Faculty of Life Sciences, University of Vienna, Vienna, Austria; ^4^Vienna Metabolomics Center, University of Vienna, Vienna, Austria; ^5^Institute of Postharvest and Food Sciences, Agricultural Research Organization, Volcani Center, Rishon LeZion, Israel

**Keywords:** pollen, heat-stress, thermotolerance, *Solanum lycopersicum*, ethylene, proteomics

## Abstract

Heat stress is a major cause for yield loss in many crops, including vegetable crops. Even short waves of high temperature, becoming more frequent during recent years, can be detrimental. Pollen development is most heat-sensitive, being the main cause for reduced productivity under heat-stress across a wide range of crops. The molecular mechanisms involved in pollen heat-stress response and thermotolerance are however, not fully understood. Recently, we have demonstrated that ethylene, a gaseous plant hormone, plays a role in tomato (*Solanum lycopersicum*) pollen thermotolerance. These results were substantiated in the current work showing that increasing ethylene levels by using an ethylene-releasing substance, ethephon, prior to heat-stress exposure, increased pollen quality. A proteomic approach was undertaken, to unravel the mechanisms underlying pollen heat-stress response and ethylene-mediated pollen thermotolerance in developing pollen grains. Proteins were extracted and analyzed by means of a gel LC-MS fractionation protocol, and a total of 1,355 proteins were identified. A dataset of 721 proteins, detected in three biological replicates of at least one of the applied treatments, was used for all analyses. Quantitative analysis was performed based on peptide count. The analysis revealed that heat-stress affected the developmental program of pollen, including protein homeostasis (components of the translational and degradation machinery), carbohydrate, and energy metabolism. Ethephon-pre-treatment shifted the heat-stressed pollen proteome closer to the proteome under non-stressful conditions, namely, by showing higher abundance of proteins involved in protein synthesis, degradation, tricarboxylic acid cycle, and RNA regulation. Furthermore, up-regulation of protective mechanisms against oxidative stress was observed following ethephon-treatment (including higher abundance of glutathione-disulfide reductase, glutaredoxin, and protein disulfide isomerase). Taken together, the findings identified systemic and fundamental components of pollen thermotolerance, and serve as a valuable quantitative protein database for further research.

## Introduction

Most crop plants are exposed to HS during some stage of their life cycle. HS, defined as the temperatures above normal optimum, is expected to become a more frequent and acute problem in the coming years (Mittler et al., 2012; IPCC, 2014; Ghatak et al., 2017b). Exposure to HS reduces yield and decreases the quality of many crops, including vegetable crops such as pepper and tomato (Kinet and Peet, 1997; Peet et al., 1998; Boote et al., 2005; Bita and Gerats, 2013). It was previously demonstrated in tomato that at daily mean temperatures of 29^o^C (32/26^o^C day/night), fruit number, fruit weight per plant, and seed number per fruit were markedly decreased compared with that at 25^o^C (Peet et al., 1998). Plants also encounter high temperature damage during spring and autumn when grown in the warmer regions of the world. During these seasons, short waves of high temperatures may be detrimental. Impaired pollen (the male gametophyte) development and functioning under high temperature conditions has been implicated in reduced yields across a large number of crop systems (Frank et al., 2009; Zinn et al., 2010; Bita and Gerats, 2013; Bokszczanin et al., 2013; Ghatak et al., 2017a). In tomato, developing pollen grains are highly HS sensitive (Pressman et al., 2002; Pressman et al., 2006). Heat-tolerant tomato cultivars (exhibiting higher yield under HS) produced larger numbers of high-quality pollen grains, exhibiting higher germination capacity, under HS compared with all tested heat-sensitive cultivars (Firon et al., 2006).

The plant male gametophyte developmental program is tightly regulated, starting inside the anther when sporogenous pollen mother cells undergo meiosis to form tetrads of cells. Each tetrad is enclosed in a thick callose wall. The microspores are released by the action of callase, an enzyme produced by the tapetum, the innermost layer of the anther that feeds the developing microspores. This developmental phase is termed microsporogenesis. The next developmental phase, culminating with the formation of a mature pollen grain, is termed microgametogenesis. During microgametogenesis, the polarized released microspores enlarge and a single large vacuole is produced (Giorno et al., 2013; Chaturvedi et al., 2016). This is accompanied by migration of the microspore nucleus to a peripheral position against the microspore wall. The microspores then undergo an asymmetric cell division (Pollen Mitosis I). The small germ cell, called the generative cell, is subsequently engulfed within the cytoplasm of the larger vegetative cell to create a novel cell-within-a-cell structure. Vegetative and germ cells have distinct fates. The vegetative cell nurtures the germ cell and gives rise to the pollen tube following successful pollination (Giorno et al., 2013; Chaturvedi et al., 2016). In tomato, during pollen tube growth in the stylar tissue, the germ cell goes through a further round of mitosis (Pollen Mitosis II) to produce twin sperm cells. Several types of metabolites accumulate in the vegetative cell during pollen maturation including carbohydrates and/or lipids, as well as starch (Firon et al., 2006; Pressman et al., 2012), along with transcripts and proteins, which are then required for rapid pollen tube growth (Pacini, 1996; Obermeyer et al., 2013). The transition from proliferating microspores to differentiated pollen is associated with a shift in gene expression (Rutley and Twell, 2015). It is thus anticipated that HS will affect pollen grains’ inherent developmental program.

Though HS is a serious problem in tomato, causing yield reduction, and though it is known that developing pollen grains (at both microsporogenesis and microgametogenesis stages) are the most sensitive to heat, the molecular mechanisms involved are not fully understood. Using high-throughput transcriptomic approaches we have identified genes that are associated with pollen HSR and may contribute to pollen heat-tolerance (thermotolerance; Frank et al., 2009). Thermotolerance is generally divided into acquired (i.e., the ability to acquire tolerance to otherwise lethal HS) and basal (i.e., the inherent ability to survive temperatures above optimal growth temperatures; Suzuki et al., 2008). Contradicting the previous notion that ‘pollen is unable to mount a significant HSR,’ our results revealed high HS regulation of members of the heat-shock protein gene family, HS transcription factors, the ROS scavenger ascorbate peroxidase and factors other than the classical HS-responsive genes (Frank et al., 2009). Remarkably, our data indicated HS induction of several ethylene-responsive genes in developing tomato pollen grains, pointing to the potential involvement of ethylene in pollen HSR. Data on the involvement of ethylene in pollen development and pollen HSR are, however, scarce (De la Torre et al., 2006; Firon et al., 2012).

With respect to vegetative tissues, evidence for the involvement of ethylene in plant thermotolerance has been reported in numerous studies. For example, pre-treatment of a cool-season grass (*Agrostis stolonifera var. palustris*) with 1-aminocyclopropane-1-carboxylate synthase (ACC; a precursor of ethylene) prior to the exposure of plants to HS increased their heat tolerance as evidenced by grass quality and leaf photosynthetic rates (Larkindale and Huang, 2004). Similarly, exogenous addition of ACC was shown to protect *Arabidopsis* seedlings against heat-induced oxidative damage (Larkindale and Knight, 2002). In addition, it was previously shown that the *Arabidopsis* ethylene-signaling mutants, *ein2* and *etr1*, are defective in basal thermotolerance (Larkindale et al., 2005). *MBF1*, a member of the transcriptional co-activator multiprotein bridging factor 1 gene family in *Arabidopsis* —MBF1c — has been previously suggested to act as a regulator of thermotolerance upstream of salicylic acid and ethylene (Suzuki et al., 2008). Recently, we have shown that ethylene is involved in maintaining tomato pollen quality under HS conditions.

The objectives of the present study were: (1) To substantiate the involvement of ethylene in tomato pollen thermotolerance (2) To contribute to our understanding of developing pollen grains’ HSR at the proteome level (3) To identify molecular pathways and specific protein candidates that participate in pollen thermotolerance.

Our results showed that developing pollen grains of tomato were highly HS-sensitive, and that pre-treating tomato plants with ethephon (an ethylene releasing compound) prior to exposing them to HS, caused a significant increase in pollen quality. In our proteomic study, we have focused on the unicellular-to-early bicellular pollen developmental stages, shown to be highly HS sensitive, and responsive to ethylene pre-treatment. The HS-responsive proteome of developing pollen grains was compared to the proteome of pollen grains kept under optimal conditions, and to that of pollen grains derived from plants treated with ethephon prior to HS exposure. The results point to an effect of HS on pollen developmental program, affecting carbohydrate and energy metabolism, representing major processes during pollen maturation. In addition, HS was found to affect proteins involved in maintaining protein homeostasis, including proteins of the translational machinery, while pre-treatment with ethephon prior to HS exposure prevented these effects. We found that a unique change in the proteome of ethephon-pre-treated pollen grains was the elevated abundance of proteins involved in maintaining cellular redox state.

## Materials and Methods

### Plant Material, Growth Conditions, Heat-stress Application and Ethephon Pre-treatment

Micro-Tom tomato (a tomato cultivar which is small, has a short life cycle and produces a relatively high number of flowers (Meissner et al., 1997) plants were grown in a temperature-controlled green-house at The Volcani Center, Bet Dagan, Israel, under natural light conditions (day length of 13–14 h) and day/night temperatures of 26/22 ± 2^o^C. The plants were exposed to HS conditions (50^o^C for 2 h) at specific flower developmental stages (detailed below). During the heat treatment, to avoid drought stress and wilting, plants were kept in a humid environment. Heat-stress was applied to plants carrying flower buds at developmental stages of 4, 3, 2, and 1 days before flower opening (A-4, A-3, A-2, and A-1, respectively). Control conditions were: keeping the plants at 25^o^C for 2 h. Following calibration of effective concentrations that had no side effects, 1 μL L^-1^ (1 ppm) ‘ethephon’ (2-chloroethyl phosphonic acid), an ethylene-releasing compound, was applied to the plants by immersing them in the solution for 18 h before exposure to HS (in the control treatment plants were treated with water without ethephon). The pH value of the ethephon solution was 4.8 (similar to the pH value of water without ethephon). Under such conditions of pH and temperature (25^o^C) ethephon is stable in solution (Biddle et al., 1976). No effect of ethephon pre-treatment on either floral or pollen development was observed. Following the treatments, the plants were kept at 26/22 ± 2^o^C day/night temperatures for at least 4 days to allow plant and flower development to continue. A scheme of the experimental design is presented in Supplementary Figure S1.

### Pollen Collection and Determination of Pollen Quality

To determine pollen quality, the developing pollen grains were allowed to mature while on the plant and pollen grains were collected at anthesis from the heat-stressed and control plants (with and without ethephon-pre-treatment). Pollen quality was evaluated immediately after pollen collection as described (Firon et al., 2012), determining the number of viable (stained purple) and non-viable (stained green) pollen grains using Alexander dye (Alexander, 1980). The number of viable pollen grains consisted of two pollen populations: germinating and non-germinating as evaluated by incubating the pollen grains in a germination solution as described (Firon et al., 2012). At least eight flowers at first day of anthesis were sampled from each treatment. Alexander dye (Alexander, 1980) was added to the germination medium after allowing for a germination time of 4 h at 25^o^C, enabling the simultaneous evaluation of the number of viable/non-viable together with the number of germinating/non-germinating pollen grains as detailed in (Firon et al., 2012). This procedure was repeated at least three times (constituting 3 biological replicates) and average results were calculated. All pollen quality results were the means of at least three biological replicates. Data are presented as mean ± SE. Multiple comparison Tukey’s HSD test was used to test for mean values that are significantly different (α = 0.05).

### Microscopy

Confocal images were captured with IX81/FV500 laser-scanning confocal microscope (Olympus Corporation, Tokyo, Japan) using 60 × 0.1 Plan Apo objectives and UV excitation filters. For confocal images, the tomato anthers were dissected to release the pollen grains. Pollen grains, released from the anthers, were stained with DAPI (4, 6-diamino-2-phenylindole) -containing buffer [0.1 M sodium phosphate pH 7, 1 mM EDTA, 0.1% Triton X-100, 0.4 μg/ml DAPI (high grade Sigma stock)]. For fluorescence microscopy, pollen samples were kept in sucrose-free germination solution (the composition of the solution is detailed in (Firon et al., 2012)), collected by centrifugation, stained with DAPI, and images were captured using Leica DNLB epi-fluorescence microscope (Germany) at 200 X magnification.

### Isolation of Pollen Grains and Collecting Leaves for Protein Extraction and Quantitative Proteome Analysis (GelC-LTQ-Orbitrap MS)

For each pollen sample, anthers collected from 60 flowers, at developmental stages A-4 and A-3, were dissected and pollen grains were obtained by slicing the anthers transversely and vortexing them in cold, sucrose-free germination solution. The solution was then filtered and pollen grains separated as detailed in (Firon et al., 2012). The released pollen grains were immediately frozen in liquid nitrogen and kept frozen at -80^o^C until use. In parallel to collecting pollen grains, leaf samples were collected from all treatment groups, collecting the first fully expanded leaves from the top-most branch, using three biological replicates.

Proteins were extracted and analyzed according to (Valledor and Weckwerth, 2014; Chaturvedi et al., 2015). Pollen samples were freeze-dried and grinded for 2 min in a shaking mill using steel balls (2 mm diameter). The homogenized pollen sample was resuspended in 200 μL of protein extraction buffer (100 mM Tris- HCl, pH 8.0; 5% SDS, 10% glycerol; 10 mM DTT; 1% plant protease inhibitor cocktail (Sigma P9599) and incubated at room temperature for 5 min followed by incubation for 2.5 min at 95°C and centrifugation at 21000 × g for 5 min at room temperature. The supernatant was carefully transferred to a new tube. Two-hundred microliters of 1.4 M sucrose was added to the supernatant and proteins were extracted twice with 200 μL TE buffer-equilibrated phenol followed by counter extraction with 400 μL of 0.7 M sucrose. Phenol phases were combined and subsequently mixed with 2.5 volumes of 0.1 M ammonium acetate in methanol for precipitation of proteins. After 16 h of incubation at -20°C, samples were centrifuged for 5 min at 5000 × g. The pellet was washed twice with 0.1 M ammonium acetate, once with acetone and air-dried at room temperature. The pellet was redissolved in 6 M Urea and 5% SDS and protein concentration was determined using the bicinchoninic acid assay. Proteins were prefractionated by SDS-PAGE. Forty micrograms of total protein were loaded onto a gel and run for 1.5 cm. Gels were fixed and stained with methanol: acetic acid: water: Coomassie Brilliant Blue R-250 (40:10:50:0.001). Gels were destained in methanol: water (40:60) and then each lane was divided into two fractions. Leaf protein extractions followed the same procedures.

### Protein Digestion and LC-MS/MS

Gel pieces were destained, equilibrated and digested with trypsin, desalted and concentrated according to (Chaturvedi et al., 2013; Chaturvedi et al., 2015; Ghatak et al., 2016). Prior to mass spectrometric measurement, the tryptic peptide pellets were dissolved in 4% (v/v) acetonitrile, 0.1% (v/v) formic acid. 10 μg of digested peptides were injected into a one dimensional (1D) nano-flow LC-MS/MS system equipped with a pre-column (Eksigent, Germany). Peptides were eluted using a Ascentis column [Ascentis Express, peptide ES-C18 HPLC column ((SUPELCO Analytical, United States), dimension 15 cm × 100 μm, pore size 2.7 μm)] during a 80 min gradient from 5 to 50% (v/v) acetonitrile, 0.1% (v/v) formic acid. MS analysis was performed on an Orbitrap LTQ XL mass spectrometer (Thermo, Germany) with a controlled flow rate of 500 nL/min. Specific tune settings for the MS were as follows: spray voltage was set to 1.8 kV; temperature of the heated transfer capillary was set to 180°C. Each full MS scan was followed by ten MS/MS scans, in which the ten most abundant peptide molecular ions were dynamically selected, with a dynamic exclusion window set to 90 s. Ion with a + 1 or unidentified charge state in the full MS were omitted from MS/MS analysis.

### Peptide and Protein Identification

Raw data were searched with the SEQUEST algorithm present in Proteome Discoverer version 1.3 (Thermo, Germany) as described in (Valledor and Weckwerth, 2014). We have used the following settings in Proteome Discoverer for data analysis which include: Peptide confidence: High, which is equivalent to 1% FDR, and Xcorr of 2, 3, 4, 5, 6 for peptides of charge 2, 3, 4, 5, 6. The variable modifications were set to acetylation of N-terminus and oxidation of methionine, with a mass tolerance of 10 ppm for parent ion and 0.8 Da for the fragment ion. The number of missed and/or non-specific cleavages permitted was 2. There were no fixed modifications, as dynamic modifications were used. The Tomato protein database was employed (Sol genomic network, iTAG2.3, which includes approx. 35,704 proteins). Peptides were matched against these databases plus decoys, considering a significant hit when the peptide confidence was high. All data are summarized in Supplementary Table S12. Descriptions of all parameters described in Supplementary Table S12, especially the creation of protein groups summarizing redundant protein identification as protein groups, can be found in the Proteome Discoverer User Guide^1^ at page 283 under the heading Protein Grouping Algorithm. All raw data and result files (annotated spectra) are further uploaded to proteomexchange^2^. Submission details are as follows; Project name: Protein Analysis of the Tomato Male Gametophyte Following Heat-stress Exposure and Pre-treatment with an Ethylene-releaser Conferring Pollen Thermotolerance. Project accession: PXD008283. The identified proteins were quantitated based on total ion count followed by a NSAF normalization strategy2:

$$\begin{array}{c} {\left(NSAF\right)}_{\kappa }={\left(PSM/L\right)}_{\kappa }/\Sigma_{i=1}^{N}{\left(PSM/L\right)}_{i} \end{array}$$

In which the total number spectra counts for the matching peptides from protein *k* (*PSM*) was divided by the protein length (*L*), then divided by the sum of *PSM*/*L* for all *N* proteins.

### Total RNA Extraction and Real Time Quantitative (q) RT-PCR

For each sample, pollen grains were isolated from anthers collected from 60 flowers, at developmental stages A-4 and A-3, as detailed above for protein extraction. The released pollen grains were immediately frozen in liquid nitrogen and kept frozen at -80^o^C until use. Pollen grains were ground to a fine powder using liquid nitrogen and sea sand (Merck, Darmstadt, Germany) and total RNA was extracted using the Tri reagent (Sigma-Aldrich, Israel). RNA was treated with TURBO DNase (AB Applied Biosystems, Ambion, CA, United States) according to the manufacturer’s instructions. Two micrograms of total RNA were used for cDNA synthesis, using Maxima first-strand cDNA synthesis kit (Thermo Scientific^3^) according to the manufacturer’s instructions. Each qPCR reaction was performed with three biological replicates, each with three technical replicates. The PCRs were performed with Platinum SYBR Green qPCR Super Mix-UDG (Invitrogen, Germany) in a Rotor-Gene 6000 cycler (Qiagen, Germany). PCR products were analyzed using Rotor Gene Series 6000 software version 1.7 (Qiagen, Germany). Values in each sample were normalized to the level of 18S ribosomal gene as a reference gene (Frank et al., 2009). All primers used are listed in Supplementary Table S13.

### Experimental Design and Statistical Rationale

Micro-Tom tomato plants were divided into groups of ten, flower-bearing plants each, and exposed to four types of treatments: Control conditions (25^o^C for 2 h), HS (50^o^C for 2 h), ethephon pre-treatment followed by control conditions and ethephon pre-treatment followed by HS (Supplementary Figure S1). This experimental procedure was repeated three times resulting in three biological replicates. Tukey’s HSD test was used for comparing pollen quality between the treatments. Proteins were extracted from developing pollen grains, isolated from 60 flowers (six flowers per each of the ten plants, constituting one biological replicate out of three) and leaves. The proteome dataset was pre-processed as described in (Valledor and Weckwerth, 2014) and data was normalized following a NSAF approach. PCA and ANOVA were performed using the statistical tool box COVAIN (Sun and Weckwerth, 2012). This software can be accessed online at http://www.univie.ac.at/mosys/software.html. For K means cluster analysis by Matlab, proteins were chosen only if they were present in all three biological replicates of at least one treatment. For functional categorization of the identified proteins we exploited the Mapman mapping file *Solanum lycopersicum*^4^. The Venn diagram was produced by Venny^5^. A protein was considered as differentially expressed between two samples if three conditions were met: (1) the protein was detected in all three replicates of at least one of the treatments (2) *p*-value for differential expression was ≤ 0.05 using *t*-test analysis or no protein was detected in any of the three replicates for one treatment (3) the fold change in protein NSAF values between the samples was at least 1.5. Differentially expressed proteins were functionally classified according to MapMan ontology (Usadel et al., 2009). Gene onthology enrichment analysis was conducted using the Plant MetGen Map (Joung et al., 2009^6^) and FDR ≤ 0.05 to indicate significant enrichment.

### Availability of the Data

The spectra of all identified pollen and leaf peptides (Supplementary Table S12) of the four treatments can be viewed online in the proteomics database proteomexchange^7^, using the following submission details: Project name: Protein Analysis of the Tomato Male Gametophyte Following Heat-stress Exposure and Pre-treatment with an Ethylene-releaser Conferring Pollen Thermotolerance and Project accession: PXD008283.

## Results and Discussion

### Application of an Ethylene Releaser to Tomato Plants Increased Pollen Quality Under Heat-Stress

**FIGURE 1:**
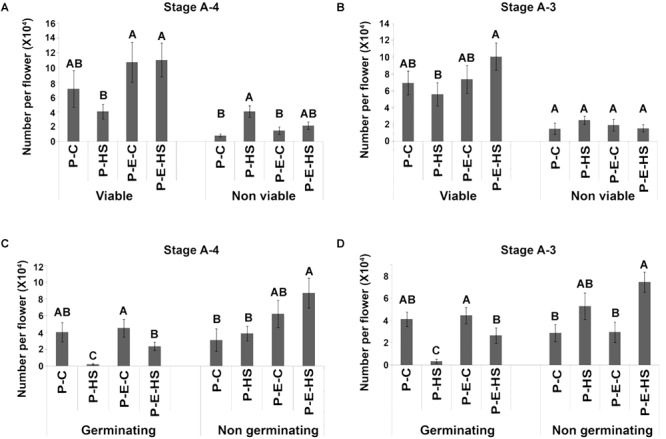
Effect of ethephon pre-treatment of tomato Micro-Tom plants on pollen quality following exposure of the plants to heat-stress. Plants were either pre-treated with ethephon (P-E-C, P-E-HS) or not pre-treated (P-C or P-HS). Heat-stress conditions were applied at 4 **(A,C)** and 3 days **(B,D)** before flower opening (stages A-4 and A-3, respectively). Mature pollen grains were collected and pollen quality determined. Data are presented as mean values ± SE (*n* = 3 biological replicates) of number of Viable, Non-viable **(A,B)**, Germinating and Non-germinating **(C,D)** pollen grains per flower (each replicate being an average of pollen derived from 8 flowers collected from different plants). **(C)** 2 h at 25^o^C; HS, 2 h at 50^o^C. In each pollen quality category (Viable, Non-viable, Germinating, Non-germinating), bars with different letters are significantly different by multiple comparison Tukey’s HSD test (α = 0.05).

Exposing tomato (*cv. Micro-Tom*) plants to HS conditions (2 h at 50^o^C) caused 42.3 and 20% decrease in the number of viable pollen grains when HS was applied at A-4 and A-3 stages, respectively (Figure 1). Pre-treating the plants with ethephon prior to HS application, prevented this decrease, resulting in 2.7- and 1.8-fold higher number of viable pollen grains, respectively, compared to non-pretreated plants (resulting in 11.0 × 10^4^ and 10.1 × 10^4^ viable pollen grains per flower, respectively; Figures 1A,B). The population of viable pollen grains consisted of germinating and non-germinating pollen grains. Pollen germination capacity, an important parameter of pollen potential for fertilization, was tested by incubating mature pollen grains in germination solution as described in ‘Experimental Procedures.’ Heat stress caused a significant decrease in the number of germinating pollen grains at all tested flower developmental stages (Figures 1C,D and Supplementary Figure S2). For all tested flower developmental stages (A-4, A-3, A-2, A-1) pre-treating the plants with ethephon resulted in an increase in the number of germinating pollen grains following HS exposure. Highest, and significant increase was observed at the earlier developmental stages A-4 and A-3: At A-4, 95 and 49% reduction in the number of germinating pollen grains was observed in non-treated and ethephon-pretreated plants, respectively, following HS, resulting in 10-fold higher number of germinating pollen grains in the ethephon pretreated plants (0.2 × 10^4^ and 2.3 × 10^4^ germinating pollen grains per flower, respectively; Figure 1C). At A-3, 92% and 46% reduction was observed in non-treated and ethephon-treated plants, respectively, resulting in more than 8-fold higher number of germinating pollen grains in the ethephon pre-treated plants (0.3 × 10^4^ and 2.7 × 10^4^ germinating pollen grains per flower, respectively; Figure 1D). Taken together, the results indicate that pre-treating the plants with ethephon caused a significant increase in the numbers of viable and germinating pollen grains following exposure to HS conditions, pointing to the involvement of ethylene in developing pollen grains’ thermotolerance. This effect was, however, stage-specific, with the earlier stages tested (A-4 and A-3) being more sensitive to both HS and ethephon treatments. It should be noted that neither HS nor ethephon applications caused a change in the leaf or the whole plant phenotype.

### Proteomic Analysis of Tomato Developing Pollen Grains Following Exposure of the Plants to Heat-Stress Conditions, With and Without Prior Treatment With an Ethylene Releaser

**FIGURE 2:**
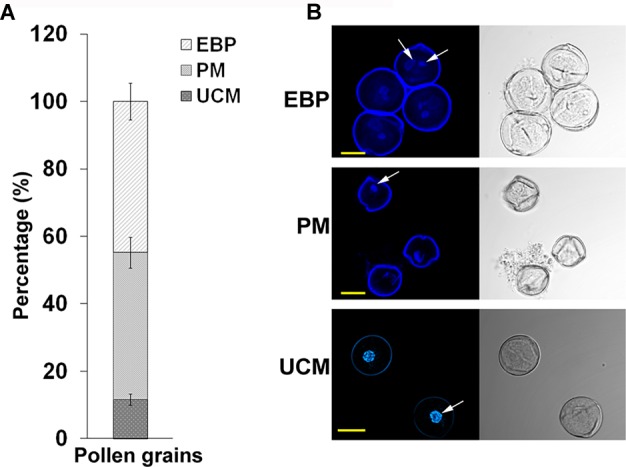
Percentage of individual pollen developmental stages in the samples used for proteome analysis and the respective confocal microscope images. **(A)** the percentage of unicellular microspores (UCM), polarized microspores (PM) and early bicellular pollen grains (EBP) in the population of A-4 and A-3 flower buds taken for proteome analysis. The percentages were calculated by fluorescence microscope at 200 X magnification using three biological replicates and the values were averaged. **(B)** all three stages of pollen development identified using confocal microscope. The arrows indicate the nucleus. Pollen grains were stained with DAPI and the images were obtained at 60 X magnification using a 0.1 NA Plan Apo water immersion objective. Scale bars, 10 μm.

Developing pollen grains were harvested from flowers at developmental stages of 4 and 3 days before flower opening (A-4 and A-3, respectively, exhibiting HS-sensitivity and responsiveness to ethephon pre-treatment) derived from *Micro-Tom* tomato plants, grown at day/night temperatures of 26/22 ± 2 ^o^C, that were exposed to short-term HS conditions (50^o^C for 2 h) following treatment with either water or ethephon. Supplementary Figure S3 relates flower developmental stages and days before flower opening to pollen developmental stages. Pollen grains were harvested immediately after HS application, using 60 flowers per each developmental stage, and pooled for proteomic analysis. The pooled pollen sample was found to consist of 11% unicellular microspores, 44% polarized microspores and 45% early bicellular pollen grains, as calculated by counting 200 pollen grains derived from combined 10 plants in each of three biological replicates (Figure 2). This population is referred to as DPGs. The following four DPGs samples were used for proteomic analysis: DPGs derived from plants maintained at 25^o^C (control conditions; P-C), DPGs derived from ethephon-pre-treated plants that were maintained at 25^o^C (P-E-C), DPGs derived from heat-stressed plants (P-HS), DPGs derived from ethephon-pre-treated plants that were exposed to HS (P-E-HS).

For comparison, proteins from young tomato leaves (taken from the top most branch of the plant) were analyzed following the same plant treatments (L-C, L-E-C, L-HS, L-E-HS samples, respectively). Three biological replicates of each treatment (from both pollen and leaves) were analyzed and separated into two fractions via SDS-PAGE prior to tryptic digestion and LC-MS/MS analysis similar to (Chaturvedi et al., 2013; Ischebeck et al., 2014). Supplementary Tables S1, S2 include all identified pollen and leaf proteins, respectively.

**FIGURE 3:**
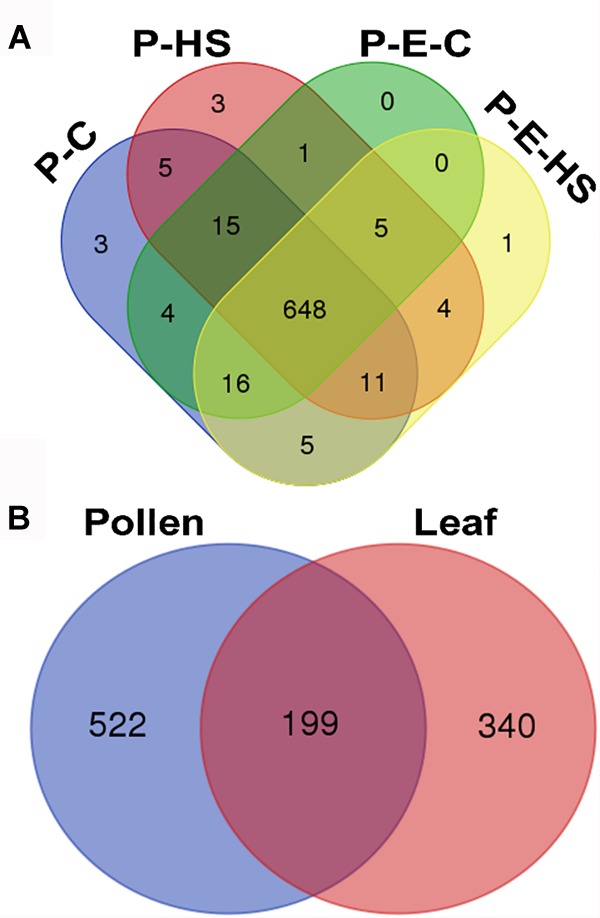
Venn diagram. **(A)** Number of proteins identified in all four pollen treatment groups. Proteins detected in all three biological replicates of at least one of the applied treatments were used. P-C, pollen derived from plants maintained at control conditions; P-HS, pollen derived from plants exposed to HS conditions; P-E-C, pollen derived from plants pre-treated with 1 ppm ethephon followed by maintaining the plants at control conditions; P-E-HS, pollen derived from plants pre-treated with 1 ppm ethephon followed by exposing the plants to HS conditions. HS, 2 h at 50^o^C. **(B)** Number of identified proteins in all pollen and leaf treatment groups. The four leaf treatment groups were the same as for the pollen samples. Proteins detected in all three biological replicates of at least one of the applied treatments were used for the analyses.

In total, 1355 proteins were identified from all four pollen samples (P-C, P-E-C, P-HS, P-E-HS; Supplementary Table S1). A total of 1064 proteins were identified from the leaf samples (L-C, L-E-C, L-HS, L-E-HS; Supplementary Table S2). The number of proteins identified in each of the pollen and leaf samples is given in Table 1. In this paper, the generated leaf proteome data set will be used to represent vegetative tissue protein profiles, to be compared with pollen protein profiles obtained at control conditions and following HS application, with and without ethephon pre-treatment. Protein abundances were quantified by peptide count and an NSAF normalization strategy (Paoletti et al., 2006). For further analyses, only proteins that were detected in all three biological replicates (‘max count 3’) of at least one of the applied treatments were considered, leading to a dataset of 721 pollen- and 539 leaf-proteins, respectively (Supplementary Tables S1, S2, respectively). A core of 648 pollen proteins (90%) was found to be common to all four treatments (Figure 3A), suggesting that main differences between the samples/treatments are manifested at protein abundance/expression levels. Of the 721 pollen proteins, 522 (72%) were not detected in any of the leaf samples (Figure 3B). These findings are similar to recently published results of protein analysis of developing tobacco pollen grains, comparing proteins from eight pollen developmental stages to proteins detected in leaves and roots (Ischebeck et al., 2014), where 65% of pollen proteins were found to be pollen-specific. Pollen transcriptomic data indicate, however, that percentage of genes that are pollen-specific is much lower, varying between 4 to 11% (Rutley and Twell, 2015). A higher proportion of pollen specific transcripts (40%) was reported by Honys and Twell (2004) for *Arabidopsis* pollen. The discrepancy for pollen-selectively expressed genes may be due to differences in the normalization methods/algorithms used, the total number of identified genes/proteins, the expression level, translational and post-translational effects, and number and diversity of sporophytic data sets used for comparison.

**Table 1:** Number of identified pollen and leaf proteins by treatment.

Treatment	Label	Number proteins
**Pollen**		
Control	P-C	1056
Heat stress	P-HS	1047
Ethephon pre-treated-Control	P-E -C	941
Ethephon pre-treated-Heat stress	P-E-HS	945
**Leaf**		
Control	L-C	693
Heat stress	L-HS	771
Ethephon pre-treated-Control	L-E -C	718
Ethephon pre-treated-Heat stress	L-E-HS	802

### Multivariate Statistical Data Mining

**FIGURE 4:**
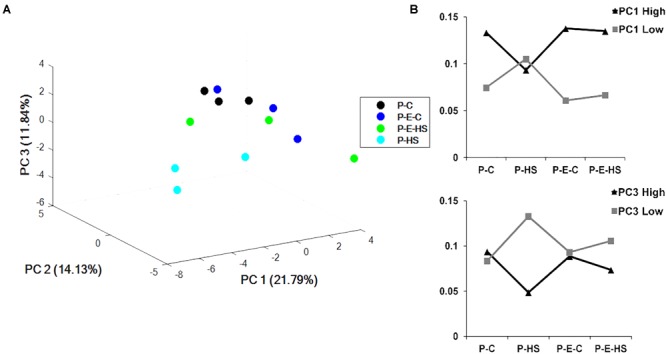
Multivariate statistical analysis. **(A)** PCA plot of the four treatments of pollen grains. **(B)** sum of the NSAF scores of the 100 highest (High) and 100 most negative (Low) loadings of PC1 and PC3, respectively. P-C, pollen derived from plants maintained at control conditions; P-HS, pollen derived from plants exposed to HS conditions; P-E-C, pollen derived from plants pre-treated with 1 ppm ethephon followed by maintaining the plants at control conditions; P-E-HS, pollen derived from plants pre-treated with 1 ppm ethephon followed by exposing the plants to HS conditions. HS, 2 h at 50^o^C.

Principal components analysis (PCA) of the protein NSAF scores was performed using COVAIN (Sun and Weckwerth, 2012). A PCA of the pollen proteins revealed that the samples could be separated into two major groups (Figure 4A), with one including only the HS-exposed microspores (P-HS), and the other including DPGs derived from the following three treatment groups: P-C, P-E-C, and P-E-HS. Thus, the separation in the PCA points to the HS-specificity of the P-HS proteome. Moreover, grouping together of the P-C, P-E-C and P-E-HS samples in the PCA, points to alterations in the heat-stressed DPGs’ proteome following ethephon pre-treatment, causing it to re-group with the ‘control’ (non-heat-stressed) samples. It should be noted that the three biological replicates of the P-HS sample are spread more than the other samples, which may be due to the sensitivity of the pollen proteome to the applied HS conditions.

The results point to high responsiveness of DPGs to HS, and to a ‘neutralizing/protective’ effect of the ethephon pre-treatment at the proteome level. The pollen proteome dataset can thus serve for identifying HS-responsive proteins, in order to come up with changes associated with the significant reduction in pollen quality, as well as looking into potential protective mechanisms activated by ethephon pre-treatment. Individual principal components data is given in Supplementary Table S3. The sum of the NSAF scores of the 100 highest and 100 most negative loadings of PC1 (Supplementary Table S3) were plotted and results are presented in Figure 4B. As PC1 differentiated/separated the samples according to treatment, both positive and negative loadings showed unique behavior of the heat-stressed sample (P-HS), with positive loadings declining and negative loadings increasing compared to all other treatments (P-C, P-E-C and P-E-HS samples; Figure 4B). Similar profiles were obtained for proteins with highest and lowest loadings in PC3, separating the P-HS treatment from the other treatments (Figure 4B). The top 100 highest PC1 positive loadings were analyzed for GO-category enrichment relative to the tomato genome database using Plant MetGenMap^8^ and found to be enriched (FDR < 0.0002) in functional terms representing processes related to carbohydrate and energy metabolism (Supplementary Table S4). The presence of a large proportion of energy related proteins has been previously described in later stages of tomato pollen development and in mature pollen (Sheoran et al., 2007; Chaturvedi et al., 2013), pointing to the need of pre-synthesized proteins for pollen germination and suggesting that proteins will be expressed/stored for functions that are in immediate need (Holmes-Davis et al., 2005). The enriched (FDR < 0.0002) GO terms in the biology process category included ‘monosaccharide metabolic process,’ ‘generation of precursor metabolites and energy,’ ‘aerobic respiration,’ ‘cellular carbohydrate metabolic process,’ ‘glycolysis,’ ‘carboxylic acid metabolic process,’ ‘acetyl-CoA biosynthesis process,’ and ‘ TCA,’ pointing to the highly metabolically active state of the DPGs. As stated above, the heat-stressed DPGs stood out in terms of the NSAF scores of the proteins involved in energy metabolism which exhibited lower values (part of them not detected at all) compared to all other treatments. These proteins included: Succinate dehydrogenase (Solyc02g093680), catalyzing the oxidation of succinate to fumarate and the reduction of ubiquinone to ubiquinol, thereby linking the TCA cycle and the electron transport system, ATP citrate lyase (Solyc05g005160, Solyc12g099260, Solyc01g059880), associated with the TCA cycle catalyzing the ATP-dependent conversion of citrate and CoA to acetyl-CoA and oxaloacetate (Sweetlove et al., 2010) as well as hexokinase (Solyc06g066440) and phosphofructokinase (Solyc07g049280), catalyzing the essentially irreversible *in vivo* inter-conversion of fructose-6-phosphate and fructose-1,6-bisphosphate (Mustroph et al., 2007). Thus, HS affects expression levels of specific proteins, by either inhibiting their transcription/translation or inducing their degradation, affecting processes crucial for cellular activity and functioning. These results may explain, at least in part, the increase in the number of non-viable pollen grains and the reduction in pollen functioning following exposure of the plants to HS conditions (Figure 1 and Supplementary Figure S2). The results thus indicate that a short heat-wave, during pollen development, may cause both pollen grains’ death and reduced capacity of the survived pollen to germinate.

The 100 most negative PC1 loadings were found to be enriched in GO terms representing cellular processes engaged in maintaining basic cellular functions, including transcription, translation and protein metabolism, as well as processes used by the cell for coping with stress conditions (Supplementary Table S4). Compared to all other treatments, the HS-DPGs stood out and showed elevated NSAF scores for proteins involved in HS-response; such as two HSP90 proteins (Solyc06g036290, Solyc10g007180), pyridoxal biosynthesis lyase (Solyc03g120090), involved in vitamin B6 biosynthesis which is a potent antioxidant (Jia et al., 2015), as well as SEC61 (Solyc02g072130), constituting the endoplasmic reticulum (ER) translocon participating in the ER quality control system to eliminate improperly folded proteins from the secretory pathway (Liu and Howell, 2010), exhibiting 1.7-, 12.1-, and 2.1-fold higher expression compared to P-C, respectively (Supplementary Table S1). The heat-stressed DPGs thus showed a HS-response and elevated expression of some protective proteins, however, this reaction was probably/eventually too late or not sufficient for protecting the DPGs as will be further elaborated below.

### Cluster Analysis

**FIGURE 5:**
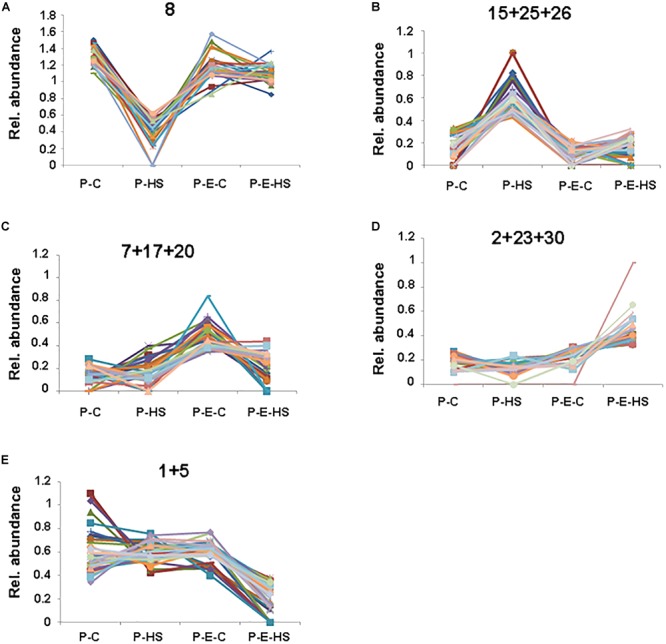
Cluster analysis. Relative abundance of proteins in a selection of groups obtained via k means clustering. NSAF scores were averaged over three biological replicates and normalized for each protein to represent their proportion of the total abundance over all four treatments. The proteins constituting each of the individual, **(A)**, or combined, **(B–E)**, clusters presented in **(A–E)** are included in Supplementary Table S5. P-C, pollen derived from plants maintained at control conditions; P-HS, pollen derived from plants exposed to HS conditions; P-E-C, pollen derived from plants pre-treated with 1 ppm ethephon followed by maintaining the plants at control conditions; P-E-HS, pollen derived from plants pre-treated with 1 ppm ethephon followed by exposing the plants to HS conditions. HS, 2 h at 50^o^C.

In order to further group the pollen proteins according to their presence in the different treatment groups, the NSAF scores were normalized for each protein and the proteins were clustered using the k means algorithm with k = 30 (Sun and Weckwerth, 2012), considering proteins present in all three replicates in at least one of the treatment groups/samples (Supplementary Table S5 and Supplementary Figure S4). In the following we discuss only selected clusters which showed a treatment-specific profile. Four clusters were found which include proteins displaying decreased or increased levels in P-HS relative to all other treatments (clusters 8 and 15, 25, 26, respectively; Supplementary Figure S4, Figure 5, and Supplementary Table S5). Proteins displaying reduced levels in P-HS (cluster 8, Figure 5A) included several 40S and 60S ribosomal proteins (RPs; Solyc06g007570, Solyc10g006070, Solyc06g083180, Solyc08g006040, Solyc07g065170 and Solyc07g009330, Solyc10g078960, Solyc06g073300, respectively), tryptophanyl-tRNA synthetase (Solyc08g074410) and eukaryotic translation initiation factor (Solyc02g089070) involved in translation, pointing to an effect of the applied HS on degradation (or on reduction in expression/translation/synthesis) of specific proteins, causing reduced levels and loss of components of the translational machinery. Since pre-treatment with ethephon prior to HS exposure (P-E-HS sample) caused more than 1.6-fold increased levels (compared to P-HS) of all these proteins (Supplementary Table S5), it is tempting to suggest that ethylene may have a role in protecting DPGs’ translation machinery. With this respect it is worthwhile mentioning that in *Brassica napus* it was shown that brassinosteroid functions to protect the translational machinery from HS by limiting the loss of some of the components of the translational apparatus (Dhaubhadel et al., 2002). Several RPs, including subunits of 40S and 60S RPs, showed HS-up-regulation compared to control conditions (Figure 5B and Supplementary Table S5), pointing to differential regulation of pollen RPs under HS. Some recent papers indicate that the expression of RPs follow stress-specific pattern and that different genes/proteins may show differential regulation, revealing a layer of regulation that may be critical for the fitness of plants under variable environmental conditions (Falcone Ferreyra et al., 2010; Hummel et al., 2012; Wang et al., 2013). It is interesting to note that almost half of the HS-down-regulated proteins in cluster 8 (46%; Figure 5A) were mitochondrial, including, for example, prohibitin (Solyc05g051510) which was suggested to play a role in mitochondrial biogenesis and protection against oxidative stress (Ahn et al., 2006). Indeed, mitochondrial proper functioning is essential for pollen development and abnormal development of the male reproductive tissues leading to male sterility was found to correlate with modifications of mitochondrial functioning (Hanson, 1991).

A subset of proteins grouped in clusters 7, 17, and 20 (Figure 5C) was expressed predominantly in ethephon-pre-treated DPGs. These proteins may be involved in the maintenance of important cellular functions as well as the regulation of pollen protective mechanisms, activating them prior to HS exposure and enabling maintenance of high pollen quality under HS. This group included proteins involved in carbohydrate metabolism and glycolysis, reproduction, cell redox homeostasis and response to abiotic stimulus (Supplementary Table S5). One example of proteins that regulate carbohydrate metabolism in plants is the 14-3-3 family that function by interacting directly with a wide range of target proteins, usually in a phosphorylation-dependent manner. Reduction or deficiency in 14-3-3 protein levels was found in male-sterile relative to male-fertile maize genotypes pointing to its important role in pollen functioning (Datta et al., 2002). A member of this family (Solyc12g010860), was found to exhibit more than 2-fold higher expression in the ethephon-pre-treated samples compared to both, control and HS samples (Supplementary Tables S1, S5), suggesting its participation in ethylene mediated pollen thermotolerance. Interestingly, a link between 14-3-3 and ethylene was recently suggested by showing that it may physically interact with different ACC synthase isoforms (Catalá et al., 2014).

Other proteins in these clusters, showing elevated expression following ethephon application (Figure 5C), included glutaredoxin family protein (Solyc06g067960), protein disulfide isomerase (Solyc05g056400) and glutathione disulfide reductase (Solyc09g091840), involved in redox homeostasis (Chi et al., 2013). Redox regulation and signaling have come into sight as crucial mechanisms able to manage critical stages during sexual plant reproduction (Traverso et al., 2013). Furthermore, it has been previously demonstrated that HS is accompanied by oxidative stress and that exogenous application of ACC protected *Arabidopsis* against heat-induced oxidative damage, thus pointing to a role for ethylene in activating mechanisms/genes that alleviate oxidative damage. Glutaredoxins are small heat-stable oxidoreductases that transfer electrons from glutathione to oxidized cysteine residues, shown to contribute to protein integrity and regulation and function in antioxidant defense (Morita et al., 2015). GSR catalyzes the reduction of glutathione disulfide to the sulfhydryl form glutathione, which is a critical molecule in resisting oxidative stress and maintaining the reducing environment of the cell. Protein-disulfide isomerase is an oxidoreductase enzyme that belongs to the thioredoxin superfamily. Taken together, these three proteins, up-regulated by ethylene, may play a role in preventing harmful effect of reactive oxygen species in DPGs under HS conditions.

Additional proteins, grouped in clusters 2, 23, and 30 (Figure 5D), expressed predominantly in ethephon-pre-treated DPGs followed by HS application, correlated with higher pollen quality under HS. This group included proteins that regulate translation, carbohydrate metabolism and response to misfolded proteins. One interesting example is a group of enzymes that play a role in carbohydrate metabolism and may be involved in signaling, regulating carbon assimilation and sugar status in plants, such as trehalose-6-phosphate synthase (Solyc10g007950, puatative; Ponnu et al., 2011) and hexokinase (Solyc06g066440; Kim et al., 2006). Hexokinase has been recently referred to as a ‘glucose sensor’ in the context of the regulation of *Arabidopsis* pollen tube glucose uptake (Rottmann et al., 2016).

The subset of proteins grouped in clusters 1 and 5 show reduced levels of specific proteins in the ethephon-pretreated sample followed by HS-exposure, relative to all other treatments (Figure 5E). This group contains proteins that function in cellular catabolic processes, including protein degradation by specific proteases and by the proteasomal ubiquitin-dependent protein catabolic process. In particular, aspartic proteinase (Solyc07g051850) as well as several components of the proteasome including SKP1 [Solyc01g111640; a core component of the SCF complex, a major type of E3 ubiquitin ligase catalyzing the last step in ubiquitin-mediated protein degradation pathway (Zhang et al., 2015)] and two 26S proteasome regulatory subunits (Solyc01g111640, Solyc05g018570) exhibited reduced abundance. Taken together, the results point to an important role of protein-homeostasis-regulation, ‘proteostasis’ (Balch et al., 2008), during pollen development, in enabling pollen proper functioning under HS.

### Proteins Involved in Protein Homeostasis Are Highly Down-Regulated Following Exposure of Developing Pollen Grains to HS

A protein was considered as differentially expressed between two samples if three conditions were met: (1) the protein was detected in all three replicates of at least one of the treatments (2) *p*-value for differential expression was ≤ 0.05 using *t*-test analysis or no protein was detected in all three replicates of one of the treatments (3) the fold change in protein NSAF values between the samples was at least 1.5. Thus, a total of 130 proteins were found to exhibit differential expression between the P-C and P-HS samples (Supplementary Table S6). Of these, 38 (29%) and 92 (71%) were found to be up- and down-regulated following HS exposure, respectively. In order to get a better understanding of the main functions affected by HS, the proteins were assigned functional categories following their annotation by the MapMan software (Thimm et al., 2004). The results indicate that the major HS-affected functional groups in tomato DPGs were protein synthesis, TCA cycle and protein degradation, all exhibiting high reduction in protein abundance (Supplementary Table S6 and Table 2). Inhibition of protein translation thus appears to be one of the earliest responses of DPGs to HS. These results are in accordance with recently published data regarding the effect of HS on vegetative tissues/cells in *Arabidopsis* [using seedlings; Echevarría-Zomeño et al., 2016) as well as in *Oryza sativa* (using suspension cells; Ueda et al., 2012)]. Furthermore, the HS effect on DPGs’ protein degradation machinery components (Supplementary Table S6) suggests that pollen cellular protein homeostasis (proteostasis) is altered. In addition, proteins involved in cellular functions, like cell cycle, cell division and transport exhibited more than 4-fold down-regulation upon HS exposure, as well as proteins involved in transcription regulation (Supplementary Table S6). High HS-down-regulation (28-fold) was observed for an RNA-binding glycine-rich protein (GRP1, solyc10g051390; Table 2). Tomato GRP1 was recently shown to be expressed in tomato fruit and was suggested to be linked to circadian processes and to function as an RNA chaperone (Müller et al., 2014). The *Arabidopsis* homologue, AtGRP8, was shown to be oxidative stress-responsive, participate in cold stress adaptation, serve as an output gene of the circadian clock and suggested as an RNA chaperone (Ciuzan et al., 2015). Data is still lacking regarding additional regulatory functions of this family of proteins. It is interesting to mention that one of the potential tomato GRP1 interactors is Solyc08g074790, eukaryotic translation initiation factor 3 subunit G-like, raising the possibility that a GRP may be involved in the regulation of translation initiation.

**Table 2:** Top 15 heat-stress down-regulated proteins in developing pollen grains.

Accession	BIN Name	Description	P-C	P-HS	P-E-C	P-E-HS	Fold change P-HS vs. P-C
Solyc10g051390	RNA.RNA binding	RNA-binding glycine-rich protein-1b	2.8	0.1	2.3	0.6	–28.1
Solyc02g068740	PS.photorespiration.glycine cleavage.H protein	Glycine cleavage system H protein 1	1.6	0.1	0.7	0.4	–16.4
Solyc02g068450	transport.p- and v-ATPases.H+-transporting two-sector ATPase	Vacuolar ATPase F subunit	1.5	0.1	0.5	1.0	–14.8
Solyc10g078960	protein.synthesis.ribosomal protein.eukaryotic.60S subunit.L21	60S ribosomal protein L21-like protein	1.3	0.1	1.3	1.0	–12.8
Solyc09g090590	development.unspecified	Calcium binding protein caleosin	1.2	0.1	0.7	0.3	–12.0
Solyc09g091170	not assigned.unknown	NADH dehydrogenase 1 alpha subcomplex subunit 13	1.2	0.1	1.5	0.1	–11.7
Solyc07g005560	protein.synthesis.initiation	Eukaryotic translation initiation factor 5A	1.1	0.1	0.8	0.1	–10.6
Solyc06g069860	Protein.synthesis.ribosomal protein.eukaryotic.60S subunit.L34	60S ribosomal protein L34	1.1	0.1	0.1	0.5	–10.5
Solyc10g007700	signaling.G-proteins	Ras-related protein Rab-2-A	1.0	0.1	0.6	0.9	–9.9
Solyc02g070310	protein.synthesis.ribosomal protein.eukaryotic.60S subunit.L32	Ribosomal protein L32	0.9	0.1	0.5	0.5	–9.5
Solyc09g007180	nucleotide metabolism.phosphotransfer and pyrophosphatases.adenylate kinase	Adenylate kinase	0.7	0.1	0.1	0.1	–7.5
Solyc04g074550	Mitochondrial electron transport/ATP synthesis.cytochrome c oxidase	Cytochrome c oxidase subunit Vib	0.7	0.1	0.1	0.9	–6.8
Solyc05g005160	TCA/org. transformation.other organic acid transformaitons.atp-citrate lyase	ATP-citrate lyase A-2	0.6	0.1	0.8	0.6	–5.9
Solyc10g018060	not assigned.unknown	Elicitor-responsive protein 3	0.6	0.1	0.1	0.3	–5.7
Solyc09g061230	redox.ascorbate and glutathione	Cytochrome b5	2.5	0.4	0.9	2.1	–5.7

*The complete dataset can be found in Supplementary Table S6. P-C, pollen derived from plants maintained at control conditions; P-HS, pollen derived from plants exposed to HS conditions; P-E-C, pollen derived from plants pre-treated with 1 ppm ethephon followed by maintaining the plants at control conditions; P-E-HS, pollen derived from plants pre-treated with 1 ppm ethephon followed by exposing the plants to HS conditions. The NSAF values were multiplied by 1000. For enabling fold-change calculation, the value ‘0.1’ was arbitrarily chosen to represent zero.*

### Ethephon Pre-treatment Causes Heat-Stressed Pollen Proteome to Be Similar to the Proteome Under Non-stressful Conditions

In order to find out to what extent the HS-response, at the proteome level, of DPGs pre-treated with ethephon (P-E-HS sample) differed from non-treated DPGs (P-HS), differentially expressed proteins between the two treatments, were assigned functional categories following their annotation by the MapMan software (Thimm et al., 2004). A total of 95 proteins exhibited differential expression between the samples (Supplementary Table S6) of these, 53 (56%) and 42 (44%) were found to be up- and down-regulated following ethephon pretreatment, respectively. The results show a general higher abundance of proteins involved in those functional groups that were highly affected by HS; namely, protein synthesis and protein degradation, TCA cycle, RNA regulation, and a number of unassigned/unknown proteins (Supplementary Table S6). For all the HS-affected proteins listed in Supplementary Table S6, including 60S and 40S ribosomal protein components, eukaryotic translation initiation factor proteins, mitochondrial import receptor subunit TOM20, 26S protease regulatory subunits, SKP1, RNA-binding proteins, and unassigned proteins, ethephon-pre-treatment caused increased protein abundance of at least 1.7-fold as compared to non-treated DPGs following HS. In addition, ethephon-pre-treatment caused modulated expression of additional components of the above-mentioned functions. For example, additional 60S and 40S ribosomal protein components and proteasome subunits exhibited more than 2.9- and 1.6-fold higher expression in P-E-HS vs. P-HS samples, respectively (Supplementary Table S6). Furthermore, ethephon-pre-treatment also showed an opposite effect compared to the HS-effect, in cases where HS caused elevation in protein abundance. For example, homologues of rRNA 2-O-methyltransferase (solyc03g025270) involved in RNA processing, ER lipid raft-associated protein (erlin-2; solyc05g012340) and histone H2A (solyc09g010400), which showed 4.1-, 4.5-, and 17.9-fold HS-elevated expression, exhibited 3.4-, 4.5-, and 4.0-fold reduction in expression following ethephon-pre-treatment (Supplementary Table S6).

Thus, the overall emerging picture is that ethephon pre-treatment caused heat-stressed pollen proteome, including the major affected components of the cellular machineries responsible for maintaining proteostasis, to become or be maintained closer to pollen proteome under control/non-stressful conditions. These results are in accordance with pollen quality results showing that ethephon pre-treatment enabled maintenance of pollen quality closer to the situation under control conditions (lower number of non-viable pollen grains and higher number of pollen that maintained germination capacity following HS exposure; Figure 1). It is noteworthy that the proteome of DPGs that were exposed ethephon-pre-treatment followed by HS show still some HS effects, which may explain the observed pollen quality results, namely, protection of pollen quality but not full protection.

Following these results, the most interesting questions are: (1) what are the mechanisms, activated by ethephon-pre-treatment, protecting protein transcription, translation, and/or degradation (enabling higher expression) under HS? (2) To what extent is/are these mechanisms selective, namely protect a specific set of proteins (3) and what is/are the regulatory factor(s) involved.

### Ethephon-Pre-treatment Causes Up-Regulation of Proteins Involved in Maintaining Cellular Redox State

Looking for potential protective mechanisms activated by ethephon-pre-treatment, differentially expressed proteins between the P-E-C and P-C samples were assigned functional categories following their annotation by the MapMan software (Thimm et al., 2004). A total of 118 proteins exhibited differential expression between the samples (Supplementary Table S6) of these, 37 (31%) and 81 (69%) were found to be up- and down-regulated following ethephon-pre-treatment, respectively. Protein disulfide isomerase (PDI; solyc05g056400) and GSR (solyc09g091840), involved in maintaining protein/cellular redox state, were among the ten proteins exhibiting most elevated abundance (>3.0-fold) following ethephon pretreatment (Supplementary Table S6). Heat stress is known to induce oxidative stress and protection against oxidative stress is an important component in plant thermotolerance (Adachi et al., 2009; Suzuki et al., 2012). There are several mechanisms/regulators that can deal with oxidative damage. Protein disulfide isomerase, a ubiquitous sulfhydryl oxido reductase found in all eukaryotic cells, is multifunctional enzyme, which catalyzes a wide range of thiol-disulfide exchange reactions, including oxidation, reduction, and isomerization, and also displays chaperone activity. In mammals and yeast, the PDIs are key protein folding catalysts activated during the ER unfolded protein stress response (Lu and Christopher, 2008). The activation of a member of this family in DPGs prior to HS exposure may thus contribute to protection of proteins’ unfolding and their subsequent degradation. It should be noted that HS also causes up-regulated abundance of this protein to a certain extent, but this may be too late for protection (Supplementary Table S6). Furthermore, it was demonstrated that ectopic expression of an *Arabidopsis* glutaredoxin (a member of the thioredoxin family) enhanced thermotolerance in tomato (Wu et al., 2012), in accordance with a 1.9-fold elevated expression of a glutaredoxin family protein (solyc06g067960) in DPGs following ethephon treatment (Supplementary Table S6). In addition, up-regulated levels of glutathione reductase, catalyzing the reduction of glutathione disulfide to the sulfhydryl form glutathione (a critical molecule in resisting oxidative stress and maintaining the reducing environment of the cell) may increase DPGs capacity for coping with the oxidative stress that accompanies HS. Ethephon application caused a 1.8-fold reduced abundance of another ROS scavenger, ascorbate peroxidase (solyc01g111510), which reduces H_2_O_2_ to water with the concomitant generation of monodehydroascorbate (Ma et al., 2008). It should be emphasized that ROS act also in pollen signaling, so there must be delicate control mechanisms(s) for managing potential oxidative damages without interfering with cellular signaling.

### Highlighting Unique Functional Categories Enriched in the Four Pollen Treatment Comparisons

**FIGURE 6:**
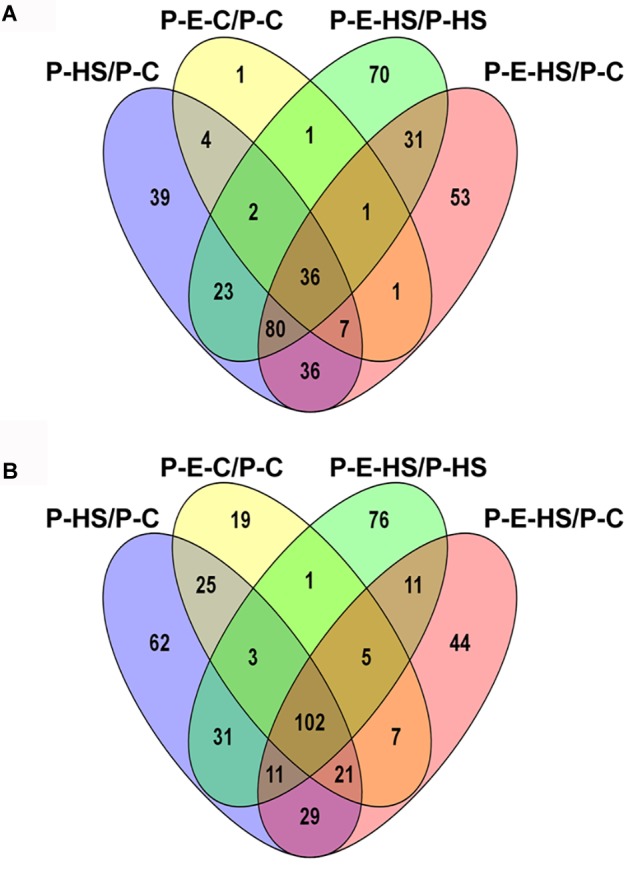
Venn diagrams of enriched GO terms in the biological process category for differentially expressed proteins between the following pollen samples: P-HS vs. P-C, P-E-HS vs. P-HS, P-E-C vs. P-C, P-E-HS vs. P-C. **(A)**, Up-regulated proteins. **(B)**, down regulated proteins. P-C, pollen derived from plants maintained at control conditions; P-HS, pollen derived from plants exposed to HS conditions; P-E-C, pollen derived from plants pre-treated with ethephon followed by maintaining the plants at control conditions; P-E-HS, pollen derived from plants pre-treated with ethephon followed by exposing the plants to HS conditions. HS, 2 h at 50^o^C.

In order to bring into focus additional pollen functions and proteins that are affected by HS, on one hand, and those that participate in the protection of DPGs from HS, on the other hand, the following analyses were undertaken. The differentially expressed proteins in the four sample/treatment comparisons (P-HS vs. P-C, P-E-HS vs. P-HS, P-E-C vs. P-C, and P-E-HS vs. P-C) were analyzed for GO-category enrichment relative to the tomato genome database using Plant MetGen Map (Joung et al., 2009) and results of significantly enriched GO terms (FDR ≤ 0.05) in the BP category (found to be most informative) are presented in Supplementary Tables S7. The data was used in order to identify unique functional terms for each comparison and results are presented in Figure 6 and Supplementary Table S8, highlighting functions and proteins that were not discussed in the previous sections.

Looking into unique functions affected by/responsive to HS application in the P-HS vs. P-C samples, thirty nine unique terms in the biological process category were found to be up-regulated (Figure 6A and Supplementary Table S8) including: ‘protein refolding’ (containing chaperonin, solyc11g069790, and HSP70, solyc04g011440 and solyc08g082820), ‘pentose-phosphate shunt’ (containing glyceraldehyde-3-phosphate dehydrogenase, solyc06g071920, mitochondrial phosphate carrier protein, solyc02g094470, transaldolase, solyc08g079070) and ‘nucleosome assembly’ (containing histone H2A, solyc11g073260, uncharacterized protein, belonging to the nucleosom assembly protein family, Solyc06g062690.2, histone H2A, Solyc09g010400). HS-up-regulation (>14-fold) of histone H2A homologues in tomato DPGs highlight the potential involvement of the nucleosome and histones in pollen HS-response. Several chromatin regulators have been shown to be involved in the regulation of stress-responsive gene networks under abiotic stress conditions. Specific histone modification sites and the histone modifiers that regulate key stress-responsive genes have been identified by genetic and biochemical approaches, revealing the importance of chromatin regulation in plant stress responses (Kim et al., 2015). Sixty two functional terms, in biological process, were found to be uniquely down-regulated (Figure 6B and Supplementary Table S8) including: ‘TCA cycle,’ ‘ribonucleotide catabolic process’ (containing elongation factor Tu, solyc06g008940, elicitor-responsive protein 3, solyc10g018060, Ras-related protein Rab-25, solyc12g010790, GTP-binding nuclear protein Ran-A1, solyc01g104680, Ras-related protein Rab-2-A, solyc10g007700, Ras-related protein Rab-18, solyc04g064510), ‘RNA processing,’ and ‘transmembrane receptor protein tyrosine kinase signaling pathway’. The results highlight the involvement of a pollen family of small GTPases in HS response. Small GTPases act as a key molecular switch for the modulation of many, plant-specific, signaling, part of them shown to participate in vesicle trafficking, pollen tube growth as well as stress response (Yang, 2002).

Looking for potential protective mechanisms activated by ethephon-pre-treatment, one unique term, ‘cell redox homeostasis,’ was detected, in the P-E-C vs. P-C comparison, among the up-regulated proteins in the BP category (Figure 6A and Supplementary Table S8). The important role in thermotolerance of protection against oxidative stress was already discussed above. Looking into unique, down-regulated, functions in P-E-C vs. P-C, 19 terms were detected (Figure 6B and Supplementary Table S8), including, ‘pyrimidine ribonucleotide biosynthetic process’ (containing adenylate succinate synthase, solyc10g080320, and dihydrolipoyllysine-residue succinyltransferase, component of the 2-oxoglutarate dehydrogenase complex, solyc07g064800), ‘mitochondrial electron transport, cytochrome c to oxygen’ (containing cytochrome c oxidase subunit VIb, solyc04g074550, cytochrome c oxidase subunit Vb, solyc12g042900) and ‘transcription’ (containing adenylate kinse, solyc09g007180, genomic DNA chromosome 5 TAC clone K21P3, solyc05g053730, prohibitin, solyc11g010190; Supplementary Table S7). The unique down-regulated functions as well as the whole list of enriched (FDR ≤ 0.05) down-regulated functions, including ‘translation,’ ‘aerobic respiration’ and ‘hexose metabolic process,’ presented in Supplementary Table S7, indicate that ethephon treatment affected the abundance of proteins belonging to house-keeping cellular pathways. Pollen quality, measured after ethephon application (Figure 1 and Supplementary Figure S2), was, however, not affected (except when applied at A-2 stage, causing increased number of germinating pollen grains). It is hypothesized that ethephon pre-treatment may cause a mild stress effect, enabling the DPGs to acquire thermotolerance, and that the activation/increased levels of proteins involved in redox homeostasis is part of the acquired thermotolerance mechanism. Similarly, Larkindale and Huang (2004) suggested that the effect of ACC treatment of *Agrostis stolonifera* was similar to the effect of acquired thermotolerance by pre-exposure to mild HS. Regarding the activation of additional protective mechanisms by ethephon treatment, especially activation of transcription factors, it is difficult to detect such molecules using proteome profiles due to their generally lower expression levels. For this reason, transcription profiling is planned.

In the P-E-HS vs. P-HS comparison, seventy unique functional terms were found to be up-regulated in the BP category (Figure 6A and Supplementary Table S8), including: ‘protein transport’ (containing trans membrane emp24 domain-containing protein 3, solyc09g082770, protein transport protein SEC31, solyc01g088020, mitochondrial import receptor subunit TOM20, solyc02g068130, MFP1 attachment factor 1, solyc04g078380; Supplementary Table S8). Up-regulation of EMP24 and SEC31 proteins points to a potential role for ER-to-Golgi trafficking in pollen heat-tolerance (Schimmöller et al., 1995; Lee et al., 2004; De Craene et al., 2014). In addition, recently it was shown that At1g18830 (SEC31A) affects pollen quality under high temperature conditions (Deng et al., 2016). It was suggested that SEC31A may confer pollen thermotolerance by either promoting the secretion of pollen coat products that confer stress tolerance, or by enhancing a trafficking function essential for pollen functioning (Deng et al., 2016). It was shown that the components of the unfolded protein response signaling pathway (including the downstream target SEC31A) play important roles in protecting male gametophyte development from HS, particularly as it relates to the interaction between developing gametophytes and the tapetum in the formation of the pollen wall. Seventy six unique functional terms were found to be down-regulated in the BP category (Figure 6B and Supplementary Table S8), including mainly cellular catabolic processes as already discussed above. In particular, ‘proteolysis,’ ‘ubiquitin-dependent protein catabolic process,’ and ‘ER-associated catabolic processes.’

In view of the presented proteome results, some interesting questions arise: At what level does ethephon pre-treatment affect gene expression, at transcription and/or post-transcriptional levels? Is there a correlation between the effect on gene expression at protein and transcript levels? In order to try and answer these questions, we chose genes involved in functions that emerge from our proteomic data as key to pollen heat-sensitivity, on one hand, and thermotolerance, on the other hand, for looking into their expression at the mRNA steady-state level.

### Comparison Between Transcript and Protein Data for Major Pollen Functions Involved in the Heat-Stress Response

**FIGURE 7:**
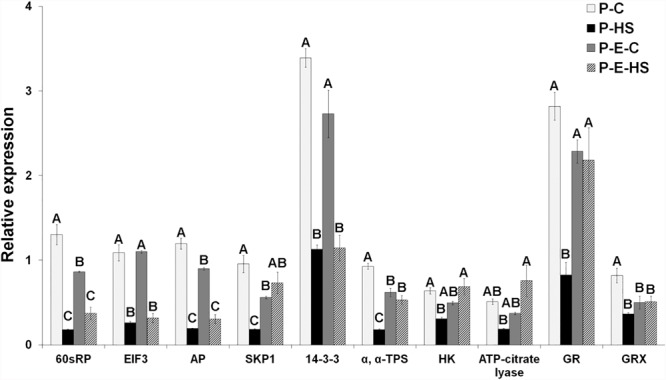
Tomato pollen transcript data for genes involved in protein synthesis and degradation, carbohydrate metabolism and signaling, TCA cycle, and redox regulation. The data was derived using qRT-PCR analyses and values were normalized relative to the expression levels of 18S rRNA in the same cDNA sample. qRT-PCR data are mean (±SE) of three biological replicates. For each gene, the bars with different letters are significantly different by multiple comparison Tukey’s HSD test (α = 0.05). Developing pollen grains were derived from: plants maintained at control conditions (P-C), plants exposed to HS conditions (P-HS), plants pre-treated with ethephon followed by maintaining the plants at control conditions (P-E-C), plants pre-treated with ethephon followed by exposing the plants to HS conditions (P-E-HS). AP, aspartic proteinase; EIF, eukaryotic initiation factor; GR, glutathione reductase; GRX, glutaredoxin; HK, hexokinase; RPs, ribosomal proteins; SKP1, s-phase kinase-associated protein 1; TPS, trehalose phosphate synthase. Corresponding Solyc numbers are given in Supplementary Table S13.

Real-time qRT-PCR analyses were performed for ten genes encoding proteins of interest in order to obtain indication on whether, or to what extent, the changes in protein abundances correlate with changes in mRNA steady-state levels. Steady-state mRNA levels for genes involved in protein synthesis (60S ribosomal protein, EIF3), protein degradation (SKP1, aspartic proteinase), carbohydrate metabolism and signaling (hexokinase, trehalose phosphate synthase, 14-3-3 protein), linking TCA cycle to other pathways like fatty acid elongation (ATP-citrate lyase), and redox regulation (glutathione reductase, glutaredoxin) were studied by on-line quantitative RT-PCR analysis using gene-specific oligonucleotide primers. The expression of each gene was normalized to that of 18S rRNA in the same sample. At transcript level, HS affected all tested genes causing more than 2-fold reduced expression, while, at protein level, HS caused reduced abundance of the majority (60%) of the genes (Solyc10g078960, Solyc05g005160 and Solyc06g066440 exhibiting > 1.5-fold reduced expression; Figure 7 and Supplementary Table S1), suggesting a significant effect of the acute HS applied on DPGs’ gene expression. Ethephon pre-treatment caused elevated expression (P-E-HS compared to P-HS), at both transcript and protein levels, for 50 and 40%, respectively, of the tested genes (including, for example, hexokinase and trehalose phosphate synthse (Figure 7 and Supplementary Table S1). Taken together, the results point to responsiveness of DPGs, at different levels of gene expression (RNA and protein), to external stimuli. Specific genes, however, like *SKP1* and *aspartic proteinase*, exhibited small changes in protein abundance across treatments, suggesting that they are tightly controlled. Indeed, tight regulation of the expression of the *SKP1* gene family in *Arabidopsis* indicates a crucial role of the ubiquitin degradation pathway during development, particularly during male gametophyte development (Marrocco et al., 2003). Protein modifications, like glycosylation, may have a role in stabilizing specific proteins, preventing or slowing down their turnover, by helping in forming long-range contacts between amino acids, which are separated in sequence and providing a more stable ‘folding nucleus.’ Indeed, both SKP1 and aspartic proteinase are known to be glycosylated (Gallardo et al., 2007; Sheikh et al., 2014). The overall expression profiles, across the four treatments, differed however, between mRNA and protein levels for most genes (eight out of ten; 80%), with only 60S ribosomal protein (solyc10g078960) and hexokinase (solyc06g066440) exhibiting similar dynamics (Figure 7 and Supplementary Table S1). Thus, different dynamics of pollen transcripts and proteins, in response to HS and hormone treatment, of genes involved in versatile cellular functions like protein synthesis, protein degradation and signaling, was observed. Performing a combined proteome and transcriptome analysis of developing *Medicago Truncatula* seeds, showed that the profiles were parallel across the time course for 50% of the comparisons made with specific processes regulated at either level. It is noteworthy that proteins involved in the regulation of protein synthesis, in oxidative stress or in detoxification displayed a profile differing from that of the corresponding transcript (Gallardo et al., 2007). In addition, ethylene-related proteins (ethylene-biosynthesis, -perception, or -signaling) were not detected in either of the pollen samples used in this study, while expression of the respective genes at transcript level was recently demonstrated by us in tomato pollen (Jegadeesan et al., 2018). This may be due to low abundance of the proteins in pollen.

### Comparing Between Pollen and Leaf Proteomes

**FIGURE 8:**
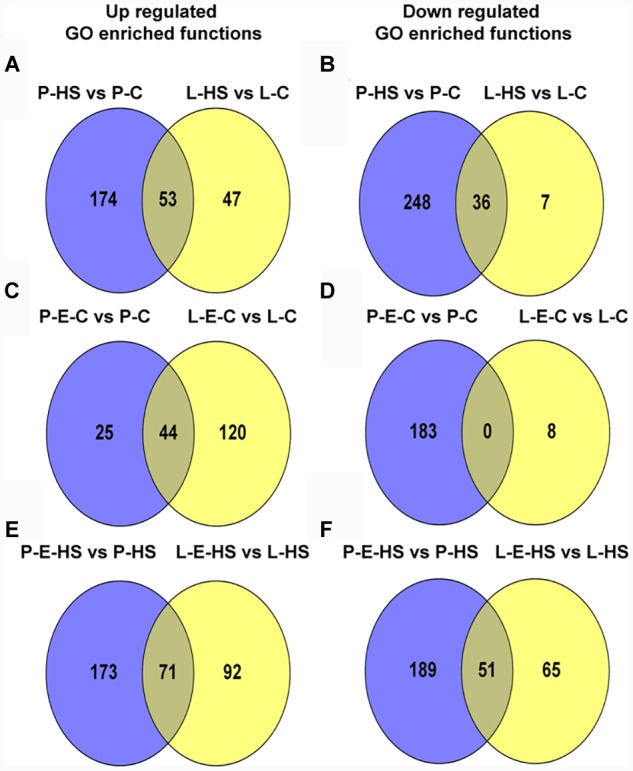
Venn diagrams comparing between pollen and leaf enriched GO functional terms in the biological process category for the four applied treatments. **A, C, E** and **B, D, F** relate to the up- and down-regulated GO terms, respectively. The following treatments were used for both leaf and pollen: HS, exposure of plants for 2 h to 50^o^C; C, 2 h at 25^o^C (control conditions); E-C, plants pre-treated with 1 ppm ethephon followed by maintaining the plants at control conditions; E-HS, plants pre-treated with 1 ppm ethephon followed by exposing the plants to HS conditions.

In order to find out whether pollen grains exhibit unique or similar responses to heat-stress and ethephon treatments, compared to vegetative tissues, pollen proteome dataset were compared to leaf proteome dataset following the same treatments. Using a dataset of 539 leaf proteins, detected in all three replicates of at least one of the applied treatments, differentially expressed proteins (exhibiting at least 1.5-fold differential expression) in the following four sample/treatment comparisons were analyzed for GO-category enrichment relative to the tomato genome database, using Plant MetGen Map (FDR ≤ 0.05; Joung et al., 2009): L-HS vs. L-C, L-E-HS vs. L-HS, L-E-C vs. L-C and L-E-HS vs. L-C. The biological process was found to be most informative (Supplementary Table S9). In order to find out to what extent the responses of the pollen and leaf proteomes to HS and ethephon applications are similar or show differences, the data was used in order to identify unique as well as common functional terms, for each comparison, between the pollen and leaf proteomes (Supplementary Tables S7, S9, respectively). High proportion of GO terms in the biological process category were found to be unique to heat-stressed DPGs (77 and 87% being HS-up- and HS-down-regulated, respectively) as compared to heat-stressed leaves (47 and 16% being HS-up- and HS-down-regulated, respectively; Figure 8 and Supplementary Table S10). These pollen functions included ‘translational initiation,’ ‘aerobic respiration,’ ‘tricarboxylic acid cycle,’ ‘ribosome biogenesis,’ ‘protein transport,’ and ‘RNA methylation’ (Supplementary Table S10). The functional terms found to be unique to heat-stressed leaves included ‘plastid organization,’ ‘photosynthesis,’ ‘chlorophyll biosynthetic process,’ as well as ‘pyruvate metabolic process,’ ‘peptide metabolic process,’ ‘gluconeogenesis’ and ‘protein homotetramerization,’ being up- and down-regulated, respectively (Supplementary Table S10). The results thus highlight major leaf functions such as photosynthesis as being HS-responsive. In particular, carbonic anhydrase (Solyc02g086820), photosystem I P700 apoprotein A1 (Solyc12g033040), chloroplastic ATP synthase delta subunit (Solyc12g056830) and fructose 1,6-biphosphatase (Solyc09g011810) exhibited more than 1.7-fold HS-up-regulated expression, while ADP-glucose pyrophosphorylase (AGPase; Solyc07g056140), lipoxygenase (Solyc01g006560), malic enzyme (Solyc05g050120), mitochondrial alpha-oxoglutarate/malate carrier (Solyc01g005620), fructose-bisphosphate aldolase (Solyc09g009260) and ribosome recycling factor (Solyc09g065270) showed more than 1.6-fold down-regulation (Supplementary Table S11). AGPase is a key enzyme of starch biosynthetic pathway, having a significant role in crop productivity and known to be HS-sensitive (Duke and Doehlert, 1996), thus reduction in protein level of any of its subunits is expected to influence starch accumulation. Fructose-bisphosphate aldolase, involved in the glycolytic pathway, was shown previously to be down-regulated at the protein level in tomato leaves subject to mild chronic HS conditions (Zhou et al., 2011). In the same study NADP-malic enzyme was up-regulated. It should be noted, that in the present study, the HS conditions applied did not show an obvious effect on either leaf or whole plant morphology and development (tested for more than 4 days after HS application). These results are in accordance with previous studies demonstrating that DPGs are the most sensitive part of the plant to HS (Firon et al., 2006; Pressman et al., 2006). It is, however, anticipated that application of a more severe HS, by either increasing the temperatures or the duration of the stress applied, would affect leaf performance (Feng et al., 2014). Looking into biological process functional terms that are affected by ethephon application, 164 and 69 GO-terms were found to be up-regulated in leaves and pollen, 73 and 36% being unique, respectively (Figure 8). The functional terms found to be unique to leaves included ‘photosynthesis,’ ‘dark reaction,’ ‘C4 photosynthesis,’ ‘response to temperature stimulus,’ ‘chloroplast organization,’ ‘carboxylic acid metabolic process,’ ‘response to cold’ and ‘response to hydrogen peroxide,’ while the terms unique to pollen included ‘reproductive process,’ ‘response to osmotic stress,’ ‘ADP transport,’ ‘ATP transport,’ and ‘cell redox homeostasis’ (Supplementary Table S9). Among the leaf proteins exhibiting differential expression following ethephon application were cpHSP70, cpHSP70-2, glutathione S-transferase-like protein, catalase, temperature-induced lipocalin, lactoylglutathione lyase, phosphoenolpyruvate carboxylase and gyceraldehyde-3-phosphate dehydrogenase (Supplementary Table S9). Antioxidant low molecular weight non-protein thiols, like ascorbate and glutathione, are important components of the non-enzymatic ROS scavenging system, while antioxidant enzymes, include superoxide dismutase, ascorbate peroxidase, catalase, and glutathione peroxidase (Larkindale and Huang, 2004; Bokszczanin et al., 2013). The results thus indicate activation of, both, non-enzymatic and enzymatic ROS scavenging systems in tomato leaves following etyhephon treatment, and confirm results in other plant systems (Larkindale and Huang, 2004). Part of the functions included exclusively in the leaf proteome, when compared to pollen proteome in the comparison L-E-HS vs. L-HS, were identical to those observed in the comparison L-E-C vs. L-C, suggesting that the effect of ethephon pretreatment consisted/remained even after HS application (Supplementary Table S10). These GO terms included: ‘photosynthesis,’ ‘response to oxidative stress,’ ‘response to temperature stimulus,’ ‘pentose phosphate shunt,’ ‘NADPH regeneration,’ ‘chlorophyl biosynthetic process,’ ‘carboxylic acid metabolic process,’ ‘pigment metabolic process’ and ‘starch metabolic process’ (Supplementary Table S10). A relatively large number of GO terms, unique to pollen grains, were detected in the down-regulated proteins following ethephon application (in both, P-E-C vs. P-C as well as P-E-HS vs. P-HS comparisons) as demonstrated in Figure 8, highlighting specific pollen functions like ‘translational initiation,’ ‘protein targeting to ER,’ ‘viral transcription’ and ‘response to peptide hormone stimulus’ (Supplementary Table S10). Taken together, the results point to pollen-specific, as well as leaf-specific responses to the applied HS conditions and ethephon application, and provide a valuable dataset of proteins to be used for further investigation into pollen and leaf thermotolerance, highlighting unique DPGs functions that are responsive to the applied treatments.

## Conclusion

The response of developing tomato pollen grains to HS conditions was analyzed and compared to proteome changes following pretreatment with an ethylene-releaser (ethephon) conferring pollen thermotolerance. In order to compare the data to results for sporophytic tissues, leaves exposed to the same treatments were also investigated. Based on the generated data, it appears that DPGs proteome is highly HS-sensitive and is responsive to the protective effect of treating the plants with an ethylene releaser.

**FIGURE 9:**
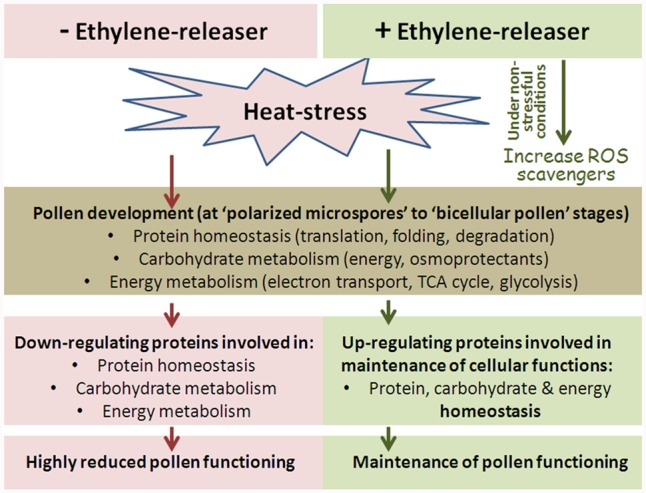
Schematic model of ethylene mediated pollen thermotolerance. Developing pollen grains at polarized microspores-to-bicellular pollen stages exhibit active metabolism, including carbohydrate and energy metabolism and active maintenance of protein homeostasis. Upon exposure to heat stress conditions, without any pre-treatment (‘-Ethylene releaser’), key biological processes in pollen development are affected, together with high reduction in pollen quality. Pre-treating developing pollen grains with ethephon (‘+Ethylene releaser’) increases ROS scavengers and causes upregulated expression of proteins involved in protein, carbohydrate and energy homeostasis following exposure to heat stress, together with maintenance of pollen quality.

The applied HS conditions were found to affect pollen developmental program, including protein homeostasis (components of the protein translation machinery and protein degradation), as well as carbohydrate and energy metabolism (Figure 9).

Interestingly, ethephon-pretreatment caused heat-stressed pollen proteome to be closer to the proteome under non-stressful conditions: A higher abundance of proteins involved in those functions that were highly HS-affected/down-regulated was apparent. These included proteins involved in protein synthesis, degradation, TCA cycle and RNA regulation (Supplementary Table S6). These results are in accordance with pollen quality results showing that ethephon pretreatment enabled maintenance of pollen quality closer to that observed under non-stressful conditions (Figure 1 and Supplementary Figures S1, S9). It should be noted that ethephon-pretreatment caused elevated expression levels of proteins known to be involved in protection against oxidative stress. Indeed, HS is known to induce oxidative stress and protection against oxidative damage is an important component of plant thermotolerance. Thus, we hypothesize that such a mechanism contributes to pollen thermotolerance, together with mechanisms enabling the maintenance of protein homeostasis at different levels, including protein translation and protein folding. In addition, we believe that the ER unfolded protein stress response may participate in pollen thermotolerance, and that ethephon treatment induces this response by, for example, increasing the expression levels of specific components like protein disulfide isomerase (Supplementary Table S6 and Figure 5C).

The comparison between DPGs and leaf proteomes, highlights unique HS-responsive pollen and leaf functions like, for example, translation initiation and photosynthesis, respectively (Supplementary Table S9). Similarly, unique functions were found for the ethylene-releaser treatment, generating a valuable database of both pollen and leaf proteins to be used for understanding networks involved in plant thermotolerance.

In order to obtain indication to what extent the changes in protein abundances correlate with changes in mRNA steady-state levels, we have looked into transcript levels of ten genes representing main functions that were found to be HS-responsive at the protein level. It was found that HS affected a large proportion of the genes at both transcript and protein levels. However, different dynamics of DPGs’ transcripts and proteins, for genes involved in versatile cellular functions (like protein synthesis, protein degradation and signaling) was observed (Figure 7).

## Author Contributions

SJ, EP, WW, and NF conceived and designed the experiments. SM acquired and designed the ethylene treatment part of the work. SJ, PC, AB, and AG performed the experiments. SJ, PC, AG, AF, and AH analyzed and interpreted the data. NF, SJ, and NR drafted the manuscript. All authors read and approved the final manuscript.

## Conflict of Interest Statement

The authors declare that the research was conducted in the absence of any commercial or financial relationships that could be construed as a potential conflict of interest.

## Footnotes

1 https://tools.thermofisher.com/content/sfs/manuals/Man-XCALI-97751-Proteome-Discoverer-21-User-ManXCALI97751-A-EN.pdf

2 http://www.proteomexchange.org

3 https://www.thermofisher.com/

4 http://mapman.gabipd.org/web/guest/mapmanstore

5 http://bioinfogp.cnb.csic.es/tools/venny/index.html

6 http://bioinfo.bti.cornell.edu/cgi-bin/MetGenMAP/home.cgi

7 http://www.proteomexchange.org

8 http://bioinfo.bti.cornell.edu/cgi-bin/MetGenMAP/home.cgi

## Abbreviations

ACC: 1-aminocyclopropane-1-carboxylate
DAPI: 4, 6-diamino-2-phenylindole
DPGs: developing pollen grains
EBP: early bicellular pollen grains
EIF: eukaryotic initiation factor
FDR: false discovery rate
GRP: glycine-rich RNA-binding protein
GO: gene ontology
GR: glutathione reductase
GSR: glutathione-disulfide reductase
HS: heat-stress
HSR: heat-stress response
NSAF: normalized spectral abundance factor
PCA: principal components analysis
PDI: protein disulfide isomerase
PM: polarized microspores
ROS: reactive oxygen species
RPs: ribosomal proteins
SKP1: s-phase kinase-associated protein 1
TCA: tricarboxylic acid cycle
UM: unicellular microspores
