# Supplementary material for: Proteomics of Heat-Stress and Ethylene-Mediated Thermotolerance Mechanisms in Tomato Pollen Grains
Source: Front Plant Sci. 2018 Nov 12;9:1558. doi: 10.3389/fpls.2018.01558 (PMC6240657; doi:10.3389/fpls.2018.01558)
Supplement: Supplementary file 14 [file Data_Sheet_1.PDF]

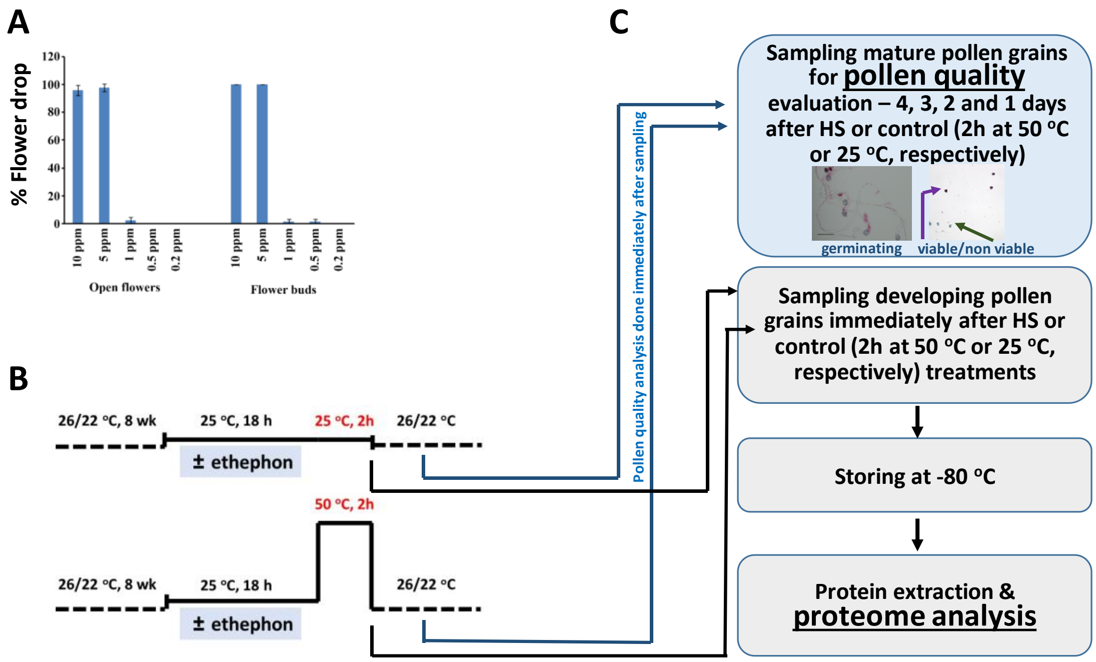

Figure S1. Experimental design. *A*, Calibrating concentrations of the ethylene-releasing substance, ethephon, to ensure minimal flower/flower-bud-drop, using tomato Micro-Tom plants. The concentration of 1 ppm, causing less than 1% flower/flower-bud-drop was chosen for all further experiments. Plans were grown for eight weeks (wk) in a temperature controlled green-house at The Volcani Center, Bet Dagan, Israel, under natural light conditions (day length of 13 – 14 h) and day/night temperatures of 26/22 ± 2 °C. *B*, Flower-bearing plants were divided into groups of ten plants each and exposed to four types of treatments: (i) '+ ethephon', plants' root system was immersed in a solution of 1 ppm ethephon for 18 h at 25 °C, prior to incubation for 2 h at either 50 °C (E-HS sample) or (ii) 2 h at 25 °C (E-C sample) (iii) '- ethephon', plants' root system was immersed in water for 18 h at 25 °C prior to incubation for 2 h at either 50 °C (HS sample) or (iv) 2 h at 25 °C (C sample). *C*, For pollen quality analyses, flowers at anthesis were sampled from plants kept 1, 2, 3 and 4 days after the 2h temperature treatment (control or HS; following the 18h incubation ± ethephon) and pollen grains were immediately collected and evaluated as detailed in 'Materials and Methods', counting the number of viable (stained purple; purple arrow), non-viable (stained green; green arrow) and germinating pollen grains (showing a pollen tube). For proteome analysis, flower-buds at developmental stage of 4 and 3 days before flower opening were sampled immediately after the 2h temperature treatment (control or HS; following the 18h incubation ± ethephon), pollen grains harvested, immediately frozen, and kept at -80 °C until protein extraction.
