# Supplementary material for: Proteomics of Heat-Stress and Ethylene-Mediated Thermotolerance Mechanisms in Tomato Pollen Grains
Source: Front Plant Sci. 2018 Nov 12;9:1558. doi: 10.3389/fpls.2018.01558 (PMC6240657; doi:10.3389/fpls.2018.01558)
Supplement: Supplementary file 15 [file Data_Sheet_2.PDF]

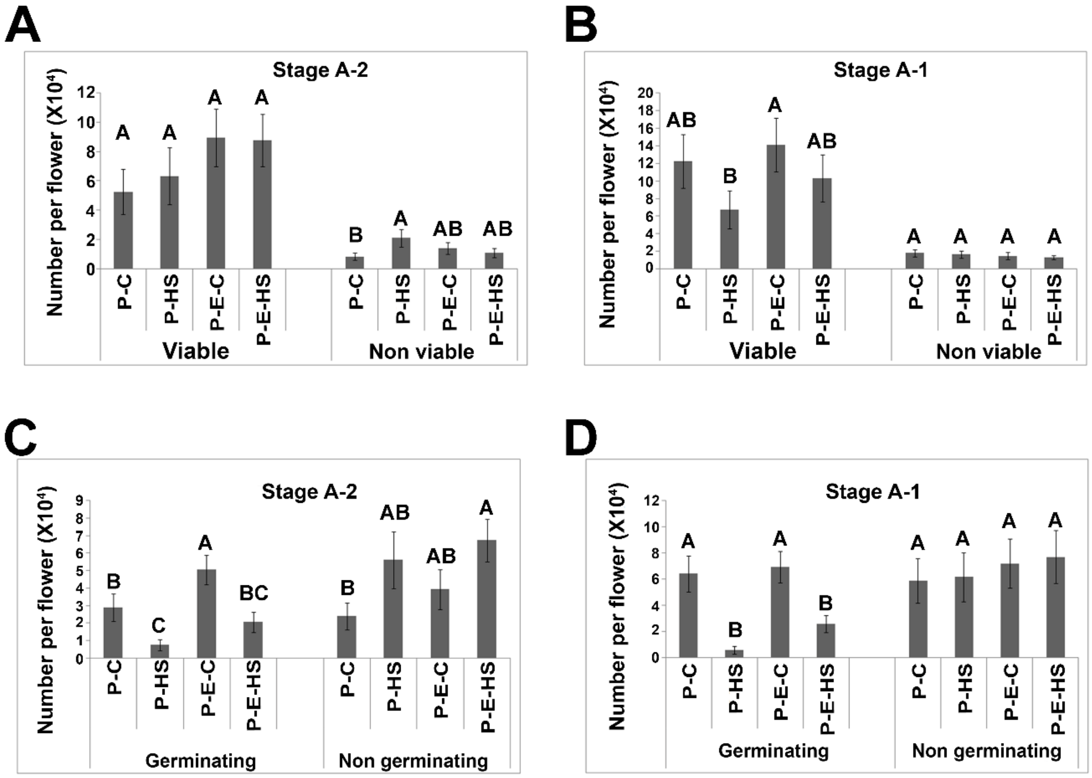

Figure S2. **Effect of ethephon pretreatment of tomato Micro-Tom plants on pollen quality following exposure of the plants to heat-stress.** Plants were either pre-treated with ethephon (P-E-C, P-E-HS) or not pretreated (P-C or P-HS). Heat-stress conditions were applied at 2 (*A*, *C*) and 1 days (*B*, *D*) before flower opening (stages A-2 and A-1, respectively). Mature pollen grains were collected and pollen quality determined. Data are presented as mean values ± SE (n = 3 biological replicates) of number of Viable, Non viable (*A*, *B*), Germinating and Non germinating (*C*, *D*) pollen grains per flower (each replicate being an average of pollen derived from 8 flowers collected from different plants). C, 2 h at 25 ^{o}C; HS, 2 h at 50 ^{o}C. In each pollen quality category (Viable, Non viable, Germinating, Non germinating), bars with different letters are significantly different by multiple comparison Tukey's HSD test ($\alpha = 0.05$).
