# Supplementary material for: Proteomics of Heat-Stress and Ethylene-Mediated Thermotolerance Mechanisms in Tomato Pollen Grains
Source: Front Plant Sci. 2018 Nov 12;9:1558. doi: 10.3389/fpls.2018.01558 (PMC6240657; doi:10.3389/fpls.2018.01558)
Supplement: Supplementary file 16 [file Data_Sheet_3.PDF]

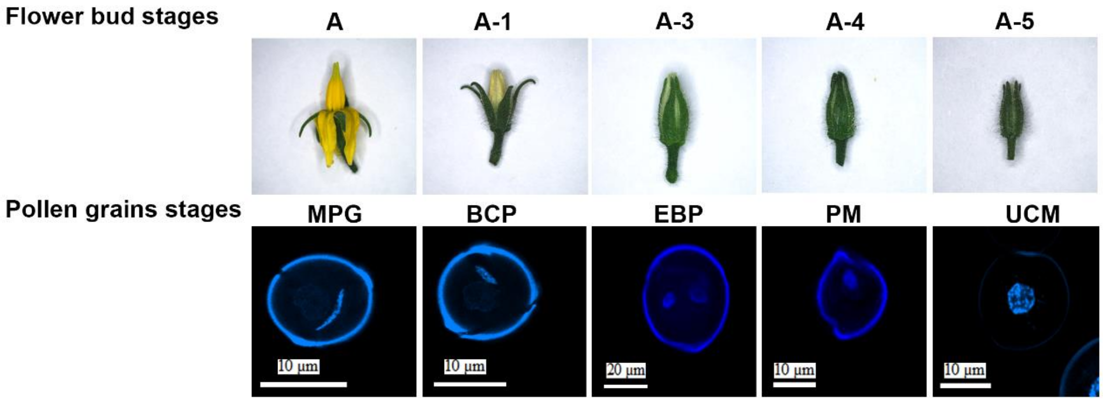

Figure S3. Tomato (Micro-Tom) flower development. Days before flower opening as related to pollen developmental stages. Microscopic pictures of pollen developmental stages represent main pollen population at the indicated flower-bud developmental stage. A-n, n days before flower opening. Pollen grains were stained with DAPI and the images were obtained at 60 X magnification using a 0.1 NA Plan Apo water immersion objective. Scale bars, 10 or 20 µm (as indicated in the picture). UCM, unicellular microspores; PM, polarized microspores (PM); EBP, early Bicellular pollen grains; BCP, bicellular pollen grains; MPG, mature pollen grains.
