# Supplementary material for: Proteomics of Heat-Stress and Ethylene-Mediated Thermotolerance Mechanisms in Tomato Pollen Grains
Source: Front Plant Sci. 2018 Nov 12;9:1558. doi: 10.3389/fpls.2018.01558 (PMC6240657; doi:10.3389/fpls.2018.01558)
Supplement: Supplementary file 17 [file Data_Sheet_4.PDF]

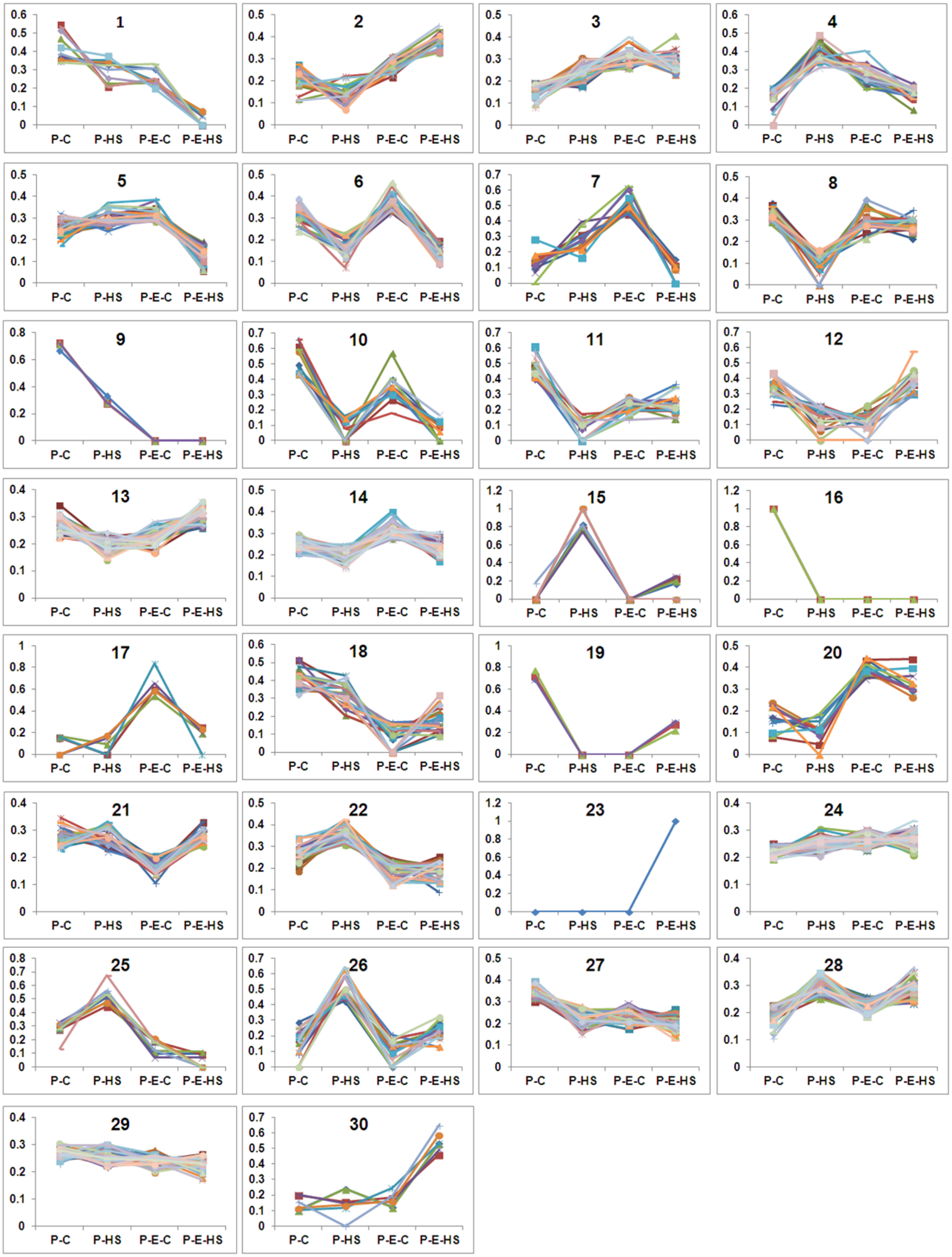
1
2
3
4
5
6
7
8
9
10
11
12
13
14
15
16
17
18
19
20
21
22
23
24
25
26
27
28
29
30
P-C
P-HS
P-E-C
P-E-HS

Figure S4. Cluster analysis. Relative abundance of proteins in 30 groups obtained by k means clustering analysis. NSAF scores were averaged over three biological replicates and were normalized for each protein to represent their proportion of the total abundance over all four treatments P-C (pollen derived from plants maintained at control conditions), P-HS (pollen derived from plants exposed to HS conditions), P-E-C (pollen derived from plants pretreated with 1 ppm ethephon followed by maintaining the plants at control conditions), P-E-HS (pollen derived from plants pretreated with 1 ppm ethephon followed by exposing the plants to HS conditions).
